# Toward the Rational Design of Tailored Ligands for Intrinsically Disordered Proteins through NMR

**DOI:** 10.1021/jacs.6c16561

**Published:** 2026-10-22

**Authors:** Cristina Ciabini, Filippo Turchi, Marco Schiavina, Tommaso Materassi, Filippo Bonini, Maria Cremonini, Francesca Clemente, Francesca Cardona, Stefano Volpi, Laura Baldini, Francesco Sansone, Roberta Pierattelli, Isabella C. Felli

**Affiliations:** † Department of Chemistry “Ugo Schiff” (DICUS), 9300 University of Florence, 50019 Sesto Fiorentino, FI, Italy; ‡ Magnetic Resonance Center (CERM), University of Florence, 50019 Sesto Fiorentino, FI, Italy; § Consorzio Interuniversitario Risonanze Magnetiche Metallo Proteine (CIRMMP), 50019 Sesto Fiorentino, Italy; ∥ Department of Chemistry, Life Sciences and Environmental Sustainability, 9370 University of Parma, 43124 Parma, Italy

## Abstract

Understanding the driving forces governing the interactions between intrinsically disordered proteins and their potential ligands represents a key step toward the rational design of drugs targeting this type of proteins. Nuclear magnetic resonance spectroscopy provides a unique opportunity to achieve detailed atom-resolved information toward this ambitious goal. We present here a set of differently functionalized calix[4]­arene-based multivalent ligands designed to target different regions of the well-characterized intrinsically disordered protein α-synuclein. Dedicated NMR experiments extended the analysis from backbone to side-chain nuclear spins, thus enabling the atom-resolved identification of short-range contacts between the calixarene ligands and methyl or aromatic groups of the protein surface, and revealing how these interactions refine the electrostatically driven association within different lysine-rich motifs. This approach provides a comprehensive description of how the macrocycles recognize and bind the target protein, offering a basis for the rational design of ligands capable of modulating protein function.

## Graphical abstract

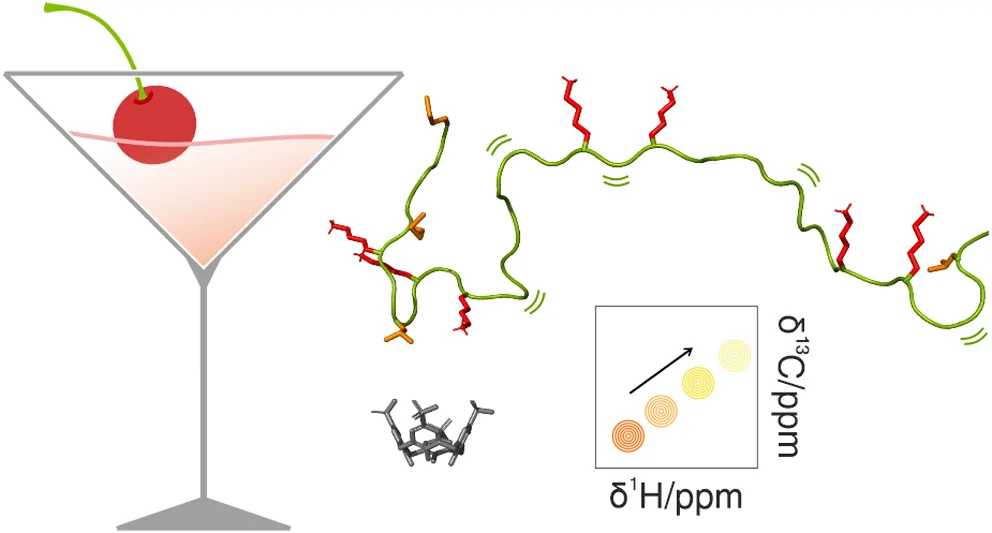

## Introduction

The importance of intrinsically disordered proteins (IDPs) has been widely recognized,^1–8^ and a number of NMR experiments have been proposed for their investigation at atomic resolution.^9–14^ These NMR techniques allow the characterization of IDPs in their native state by revealing regions characterized by secondary structural propensities^15–17^ as well as compact states through ^15^N backbone relaxation^18^ and paramagnetic relaxation enhancement (PRE) experiments.^19,20^ It is now important to shift our attention to the investigation of the modalities through which IDPs interact with biological partners, such as proteins, nucleic acids, lipids, biological membranes, metal ions, metabolites, as well as other exogenous ligands.^21–24^ These can be very different from the widely investigated interactions involving globular proteins.

Most of the NMR studies performed to date on IDPs have focused on the investigation of backbone resonances through 2D ^1^H–^15^N HSQC experiments. The development of ^13^C-detected experiments has expanded the investigation to conditions where amide proton resonances are broadened beyond detection, or extensive overlap in 2D NMR spectra limits conventional approaches, enabling the characterization of IDPs in a variety of different conditions, also approaching the physiological ones.^13,25,26^ NMR studies of IDPs remain largely restricted to the protein backbone. Notable exceptions include the investigation of carboxylate and carbonyl-containing amino acid side chains through ^13^C-detected NMR experiments, which, as an example, revealed how α-synuclein senses calcium ion concentration changes through carboxylate groups of aspartate and glutamate residues clustered in its C-terminal region.^27,28^

We propose herein to focus on amino acid side chains, which play a central role in determining protein properties and mediating interactions with other proteins, nucleic acids, and ligands. Despite their functional importance, side chains have been sparsely investigated in IDPs. This is largely due to the extreme resonance overlap, which becomes increasingly pronounced when moving away from the backbone as a result of the disorder typical of IDPs.

Here, we present a set of NMR experiments to investigate key resonances of amino acid side chains with particular emphasis on methyl groups and terminal lysine side chains. The conventional approach to monitor side chains for globular proteins is largely based on the use of HCCH-TOCSY NMR experiments for the assignment of side chain nuclear spins.^29,30^ This approach is bound to fail for IDPs because of the extreme resonance overlap for amino acids of the same type. In addition, when studying globular proteins, 2D NMR experiments are often optimized for sensitivity rather than for resolution, whereas the investigation of IDPs requires experimental designs that prioritize resolution. The novel experiments we propose exploit the favorable relaxation properties of IDPs that permit long magnetization transfer pathways and extended acquisition times, thereby maximizing spectral resolution and enabling analysis of a wide range of aliphatic and aromatic side chains.^31,32^

This approach has been further demonstrated by characterizing the interactions of α-synuclein with a series of multivalent ligands designed to target distinct regions of the protein in its disordered state, providing detailed insights into the molecular determinants of ligand recognition.

Human α-synuclein is an intrinsically disordered protein highly expressed in presynaptic terminals in the central nervous system.^33–35^ Its 140 amino acid sequence (Figure 1, panel A) can be divided into three main regions: the N-terminal region (M1-K60), enriched in positively charged amino acids, the NAC (Non Amyloid Component) region (E61-V95), which is relatively hydrophobic and significantly contributes to α-synuclein aggregation, and the C-terminal region (K96-A140), which is rich in negatively charged amino acids, proline and tyrosine residues. This protein is involved in several neurodegenerative disorders, known as synucleinopathies, including Parkinson’s disease (PD).^1,36–38^ These disorders are characterized by the accumulation of α-synuclein amyloid deposits in brain tissues. However, diverse cellular environments (variations in cell type, lipid composition, proteostasis, and oxidative stress) alter protein folding and degradation, yielding different pathological outcomes and disease subtypes. This variability, together with the elusive, chameleonic nature of α-synuclein, renders the identification of strategies to prevent these pathologies very challenging, and no molecules capable of preventing aberrant amyloid formation are available for clinical applications.

**Figure 1:**
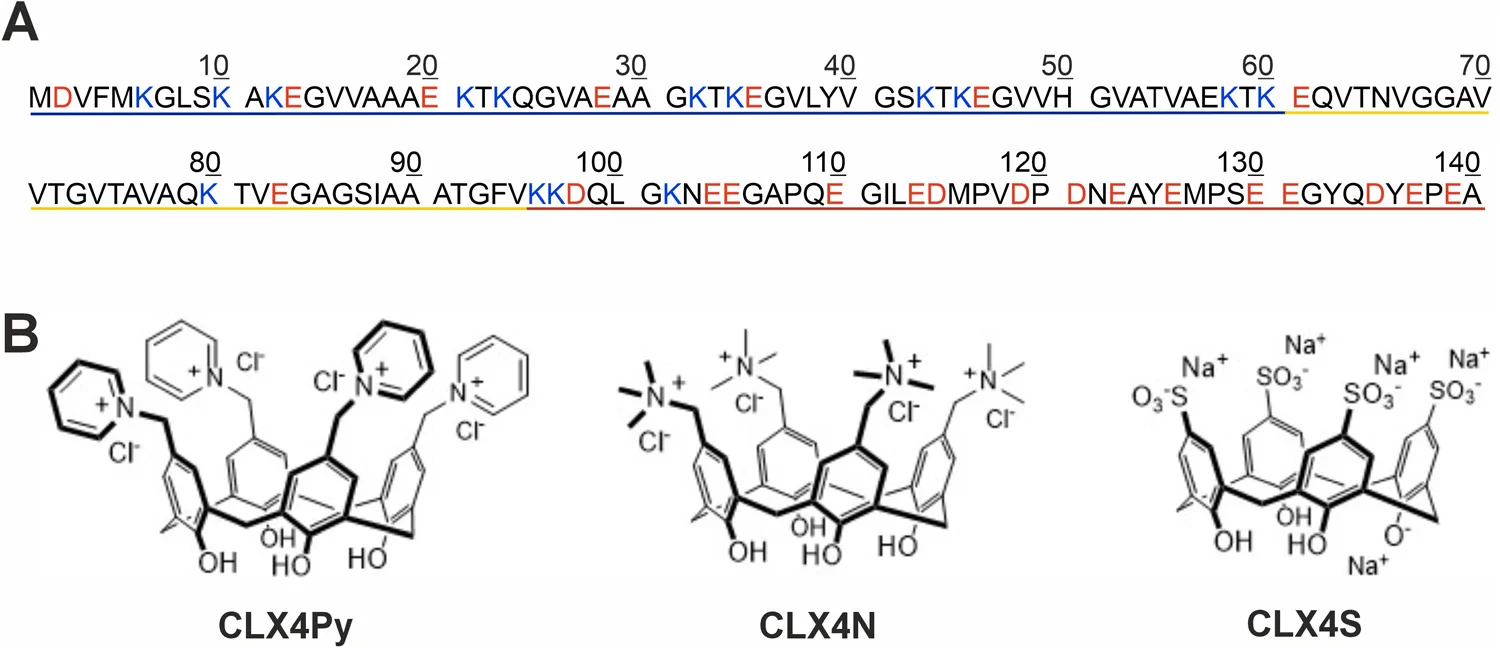
Panel A shows the primary structure of α-synuclein. Positively and negatively charged amino acids are color-coded in blue and red, respectively. The three major regions of α-synuclein are underlined with different colors: N-ter (1–60) in blue, NAC (61–95) in yellow, C-ter (96–140) in red. Panel B shows chemical structures of the three differently functionalized calix[4]­arenes investigated in this study.

Calixarenes are versatile macrocyclic receptors that have emerged as powerful tools for recognizing protein surfaces and modulating their structure and function.^39,40^ Binding site selection has been achieved either by decorating their rims with peptide- ^41–43^ or carbohydrate-based^44,45^ targeting units directed toward predefined epitopes or by introducing simpler functionalities that allow the ligands to probe the biomolecular surface for complementary regions. The latter strategy has proved particularly effective with anionic calixarenes bearing sulfonate or phosphonate groups, which have been employed to promote protein crystallization and direct protein assembly into porous frameworks.^46–49^ These applications have predominantly been explored with globular proteins, and tailored NMR experiments were proposed to focus on lysine and arginine side chains.^50,51^ They were recently used to provide information on the disordered N-terminal tail of a globular protein^52^ through lysine or methionine encapsulation into a tetrasulfonated calix[4]­arene, with the templating effect of the macrocyclic receptor inducing a transition toward more ordered conformations in neighboring residues.^52^ Ligand-mediated assembly and disorder-to-order transitions have also been observed with other supramolecular receptors, including cucurbiturils^53^ and molecular tweezers.^54^ Beyond these structural effects, macrocyclic ligands have been reported to modulate the biological activity of IDPs. Indeed, peptide-functionalized, sulfonated, and phosphonated calix[4]­arenes have shown promising ability to inhibit amyloid aggregation in cellular models of Alzheimer’s disease,^55,43,56^ demonstrating that their peculiar recognition properties remain effective in complex biological environments.

In this context, elucidating the mechanisms underlying IDP recognition by macrocyclic ligands is crucial for their rational design. Here, we propose a novel suite of NMR experiments that provides atom-resolved information on backbone and side chain nuclear spins to investigate how calixarene upper-rim functionalization governs the recognition of distinct α-synuclein regions. These studies, involving cationic and anionic calixarenes, revealed a recognition pattern in which charge complementarity directs the macrocyclic ligands toward protein regions of opposite surface charge. In addition, detailed analysis of the binding of tetrasulfonatocalix[4]­arene to positively charged regions further revealed a complex interplay among direct ionic contacts and hydrophobic effects. Lysine recognition likely represents the primary binding interaction, while hydrophobic residues provide additional stabilization.

## Results

### Different Functions on the Calixarene Scaffold to Target Different Regions of α-Synuclein

Three calix[4]­arene-based ligands were selected for this study (Figure 1, panel B). In all three compounds, the macrocyclic scaffold, composed of four aromatic rings linked by methylene bridges, is unsubstituted at the lower rim, allowing the phenolic hydroxyl groups to establish a network of intramolecular hydrogen bonds, further reinforced by the deprotonation of one of the OH groups at the experimental pH.^57^ This hydrogen-bonding network locks the four phenolic units in the same orientation, giving rise to the characteristic cone conformation of the macrocycle. As a result, the hydrophobic cavity is rigid and preorganized, and is available for the inclusion of small portions of guest molecules through π–π, CH−π, and cation−π interactions.^56,58–60^ By contrast, the upper rim was functionalized with three different substituents to impart distinct recognition properties to the ligands. **CLX4S** ^61^ bears sulfonate groups, which confer an overall negative charge, whereas **CLX4N** ^62^ and **CLX4Py** are functionalized with permanently cationic groups. Specifically, **CLX4N** carries trimethylammonium substituents, while **CLX4Py** contains pyridinium groups, which provide an additional aromatic surface. **CLX4S** and **CLX4N** were prepared following literature procedures,^61^ ,^62^ while **CLX4Py** was synthesized from a tetrachloromethyl precursor^63^ through nucleophilic substitution in neat pyridine (see the Experimental Section).

The interaction of the three ligands with α-synuclein was followed through a series of 2D HN correlation NMR spectra collected throughout a titration at different protein/ligand molar ratios. Figure 2 shows two spectral regions for each NMR titration, revealing gradual chemical shift perturbations as a function of ligand concentration, typical of a fast exchange regime. The magnitude of the spectral changes was already significant at the first titration point, corresponding to a 1.0:0.4 α-synuclein:ligand molar ratio, and was much larger than the one observed in studies involving the same protein and other types of aromatic ligands.^28,64–67^ The larger chemical shift perturbations compared with previously reported ligands enabled straight forward and unambiguous mapping of the protein regions preferentially involved in calixarene binding.

**Figure 2:**
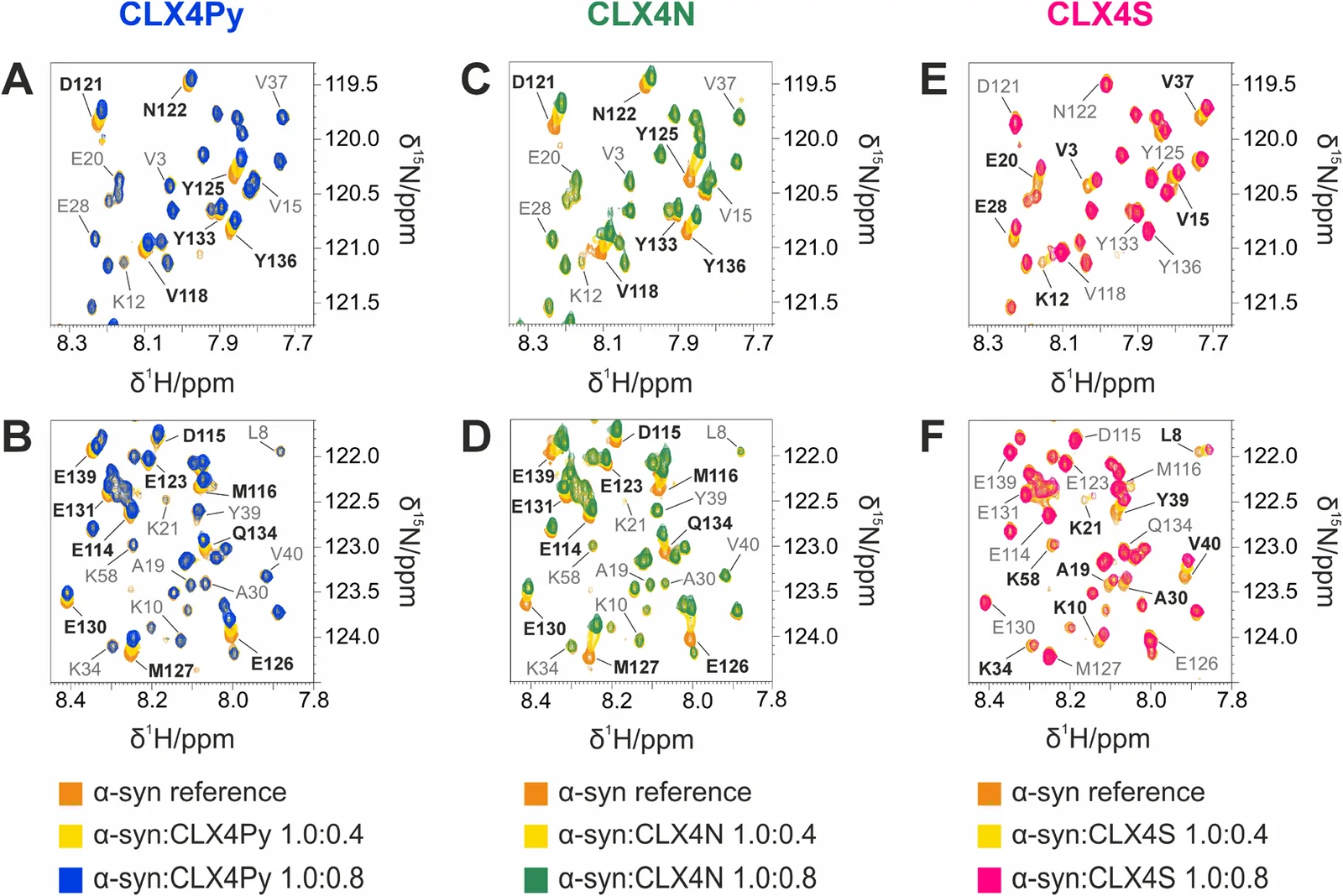
Selected regions of 2D HN NMR spectra (2D ^1^H–^15^N BEST TROSY) acquired on samples of 200 μM α-synuclein in 25 mM TRIS, 25 mM KCl, pH 7.2, upon addition of the three selected ligands: **CLX4Py** (panels A and B), **CLX4N** (panels C and D), and **CLX4S** (panels E and F). In each panel, the reference spectrum (orange) is overlaid with spectra recorded at α-synuclein:ligand molar ratios of 1.0:0.4 (yellow) and 1.0:0.8 (blue, green, and magenta for **CLX4Py**, **CLX4N**, and **CLX4S**, respectively). Assigned residues are labeled within the spectra: residues whose assignment is reported in black are the ones that undergo chemical shift perturbations upon ligand addition, while residues whose assignment is reported in gray remain unaffected. The spectra were acquired at 298 K on an instrument operating at 950 MHz (^1^H Larmor frequency) to follow the interaction with **CLX4Py** and **CLX4S** and on an instrument operating at 900 MHz (^1^H Larmor frequency) to follow the interaction with **CLX4N**.

A closer analysis of the specific changes induced by the calixarenes reveals that each ligand selectively affects distinct sets of cross-peaks, depending on its upper rim functionalization.

As shown in Figure 2 (panels A, B, C, and D), the two cationic ligands **CLX4Py** and **CLX4N** interact with residues in the C-terminal region of α-synuclein comprising amino acids E104-A140, rich in negative charges due to the presence of aspartate and glutamate residues. Very similar effects are observed upon addition of the two ligands, with the same set of residues experiencing chemical shift changes. The main difference lies in the magnitude of the chemical shift perturbations, which, together with line broadening, are more pronounced for **CLX4N** than for **CLX4Py**. Although larger spectral changes do not necessarily indicate higher affinity for the target protein, these observations suggest that upper-rim trimethylammonium groups, bearing localized positive charges, may favor recognition of the C-terminal region of α-synuclein over aromatic pyridinium groups with delocalized charges. More pronounced effects are observed around residues Y125 and E137. The anionic ligand **CLX4S**, instead, interacts with the N-terminal region of the protein, rich in positive charges mainly provided by lysine residues, causing more pronounced effects around residues K11 and Y39. A completely different set of cross peaks is perturbed upon addition of this ligand (Figure 2, panels E and F) if compared to the case of cationic ligands (**CLX4N** and **CLX4Py**).

A comprehensive view of the different binding localization of the three ligands with α-synuclein can be obtained by reporting, for each titration point, chemical shift perturbations (CSPs) as a function of the residue number. The CSP profiles shown in Figure 3 clearly highlight how the different functionalization of the calixarene upper rim directs the ligand toward distinct regions of α-synuclein.

**Figure 3:**
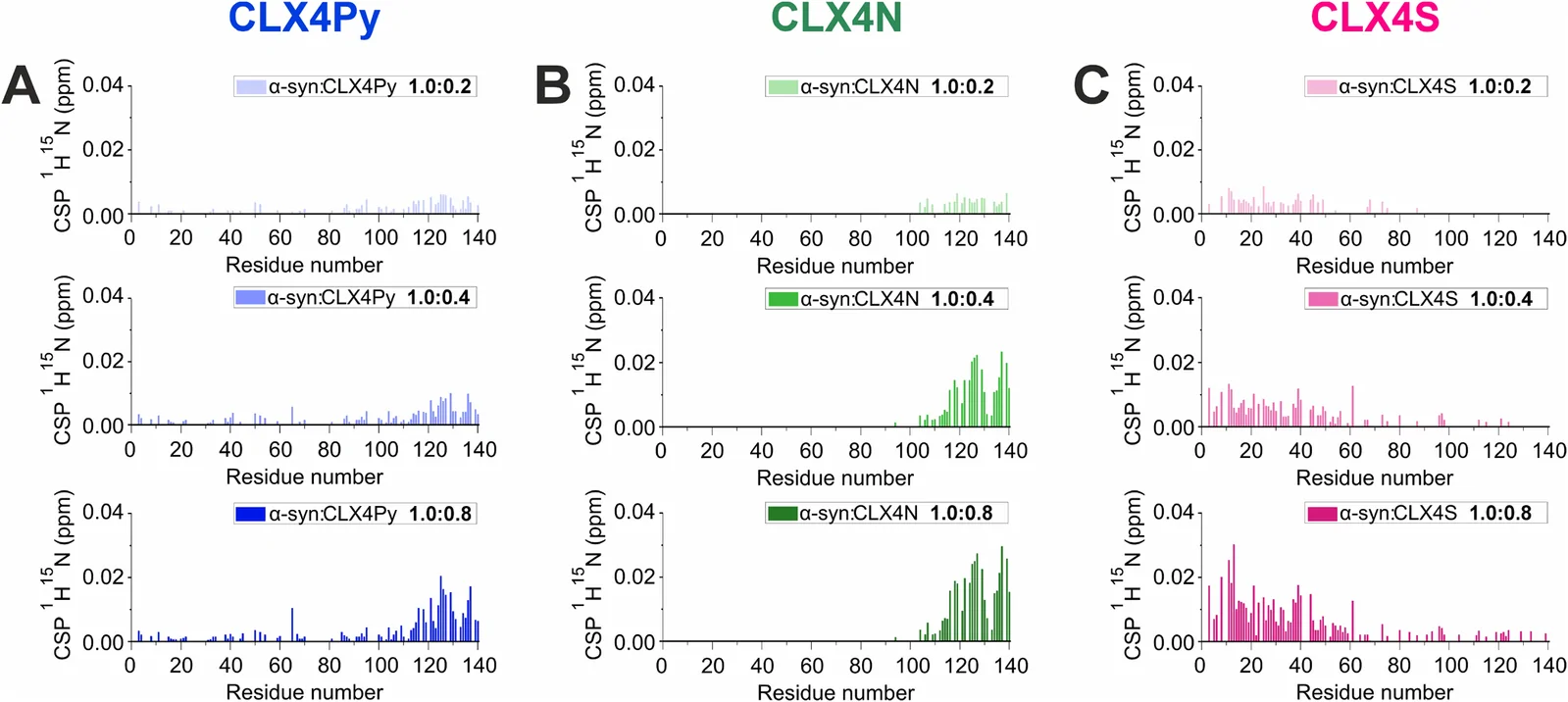
Plots of ^1^H–^15^N chemical shift perturbation (CSP) values against the residue number. The three plots report the values obtained comparing the chemical shift of 200 μM free α-synuclein with the ones available at 1.0:0.2, 1.0:0.4, 1.0:0.8 α-synuclein:ligand molar ratio in blue (A), green (B), and magenta (C) for **CLX4Py**, **CLX4N**, and **CLX4S**, respectively.

The observed trends are consistent with the heterogeneous charge distribution along the α-synuclein sequence. These observations suggest that charge–charge electrostatic interactions play a key role in ligand recognition and binding of intrinsically disordered proteins. However, not all charged amino acids are perturbed to the same extent and neutral amino acids are also affected by the presence of the ligands, indicating that additional contributions are important in determining the interaction sites of the tested calixarenes.

### Zooming into the α-Synuclein–CLX4S Interaction

To characterize more in detail the recognition process between α-synuclein and this type of macrocyclic ligand, we focused on the negatively charged derivative **CLX4S** by recording an extended NMR data set. As a first step, an additional titration was performed, acquiring 2D CON^68^ and 2D CACO^27,^ ^69^ experiments. Since the 2D CON and 2D CACO experiments are insensitive to solvent exchange broadening, the intensity of all of the peaks is less heterogeneous in the spectra; in addition, the CON spectrum allows resolving most of the cross peaks and accessing fine details about the interaction. Their combined use allows the detection of all of the resonances, thus providing a clearer picture of the binding phenomenon.

Figure 4 reports selected regions of CON spectra with some of the most perturbed residues; a complete plot of the CSP observed is reported in the Supporting Information together with the analogous data obtained through 2D HN spectra (Figure S1). The superimposition of the entire CON spectra is reported in Figure S2. Consistent with the previously observed behavior, these data confirm a preferential interaction of **CLX4S** with the positively charged N-terminal domain of α-synuclein; experiments acquired at higher ionic strength show slightly weaker CSP effects, although the interaction sites are maintained (Figure S3, panels A and B). Lysine residues are significantly perturbed as previously reported in the literature for globular proteins.^46,51^ However, not all lysine residues within the N-terminal domain respond to the ligand to the same extent. Distinct lysine residues exhibit substantial chemical shift changes, whereas others show only minor perturbations. This behavior indicates that the interaction is not only driven by the overall positive charge of the N-terminal domain, as one could expect for a highly disordered protein in which amino acid side chains are largely solvent exposed, but also exhibits a residue-specific selectivity. Analysis of the CACO NMR spectra supports this interpretation and further reveals that the negatively charged carboxylate groups of aspartate and glutamate residues (Figure S4) are largely unaffected by **CLX4S**. The only exception is D2, which is perturbed upon CLX4S addition, possibly because of its proximity to the solvent-exposed N-terminal amino group.

**Figure 4:**
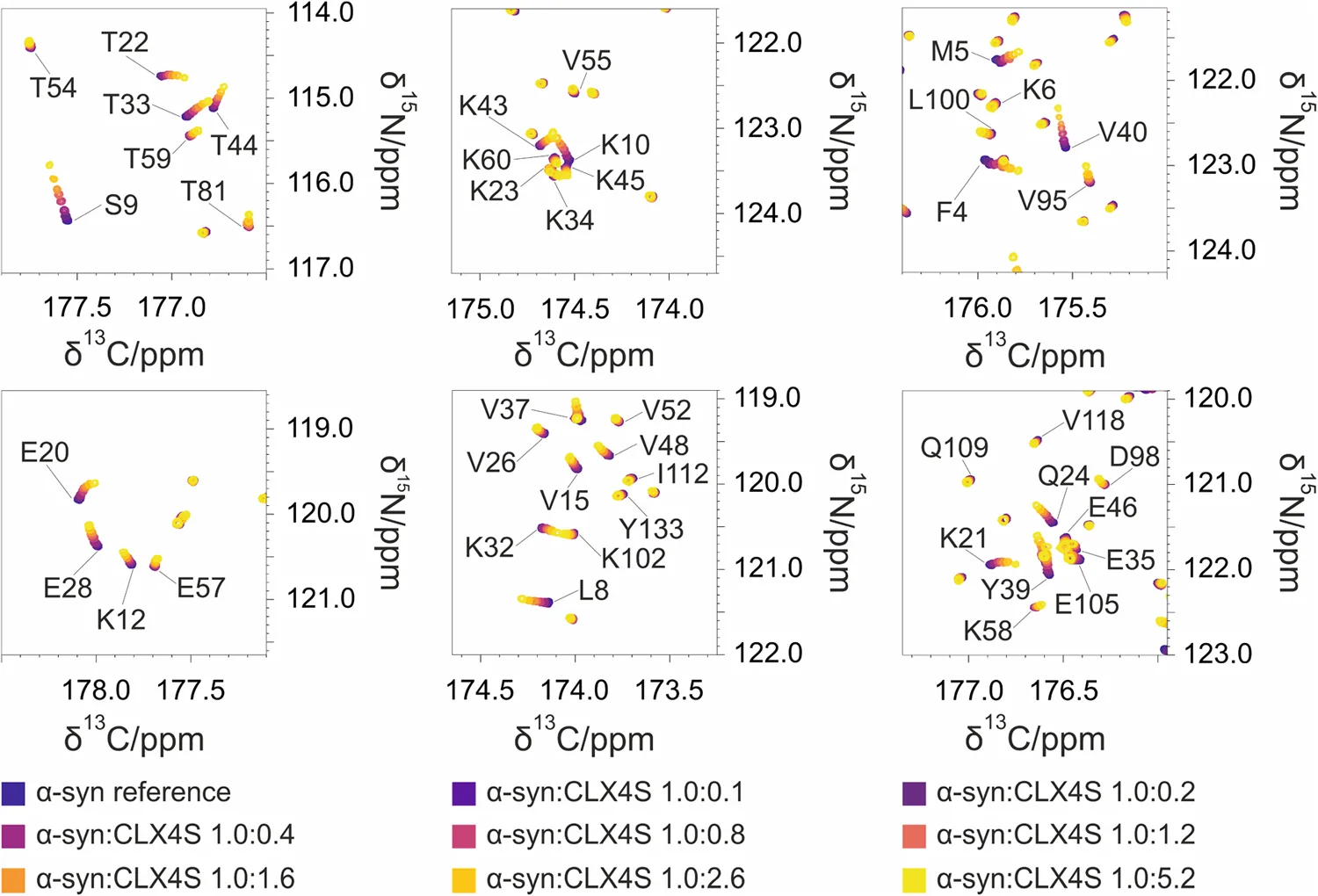
Selected regions of the 2D ^13^C–^15^N CON spectra acquired upon addition of CLX4S to a solution containing 310 μM α-synuclein. Each panel displays a different spectral region and shows the overlay of the reference spectrum of α-synuclein (blue) with spectra acquired at α-synuclein:**CLX4S** molar ratios of 1.0:0.1 (dark violet), 1.0:0.2 (magenta), 1.0:0.4 (dark pink), 1.0:0.8 (pink), 1.0:1.2 (coral), 1.0:1.6 (orange), 1.0:2.6 (light orange), 1.0:5.2 (yellow). Key residues undergoing chemical shift perturbations are labeled within the spectra by reporting the ^15^N chemical shift assignment. The spectra were acquired at 298 K on an instrument operating at 1200 MHz (^1^H Larmor frequency).

Figure 5 illustrates representative examples of CSP obtained from the CON spectra as a function of ligand concentration for three residues located in different regions of α-synuclein that experience significant shifts upon the addition of the ligand. Residue S9, which was not visible in 2D HN spectra due to exchange processes with the solvent, and residue V40, both located in the N-terminal region, show a progressive increase in CSP from the earliest points of the titration, resulting in a linear chemical shift trajectory of the cross peaks across the explored concentration range. In contrast, residue V95 remains largely unaffected at low ligand concentrations and starts displaying measurable perturbations only at higher α-synuclein:**CLX4S** molar ratios (starting from 1.0:0.8). Under the assumption of a 1:1 interaction, apparent *K*_d_ values of 740 ± 60 μM and 550 ± 40 μM were determined for residues S9 and V40, respectively (Figure S5); for V95, *K*_d_ values could not be determined with this simple assumption from the NMR experiments.

**Figure 5:**
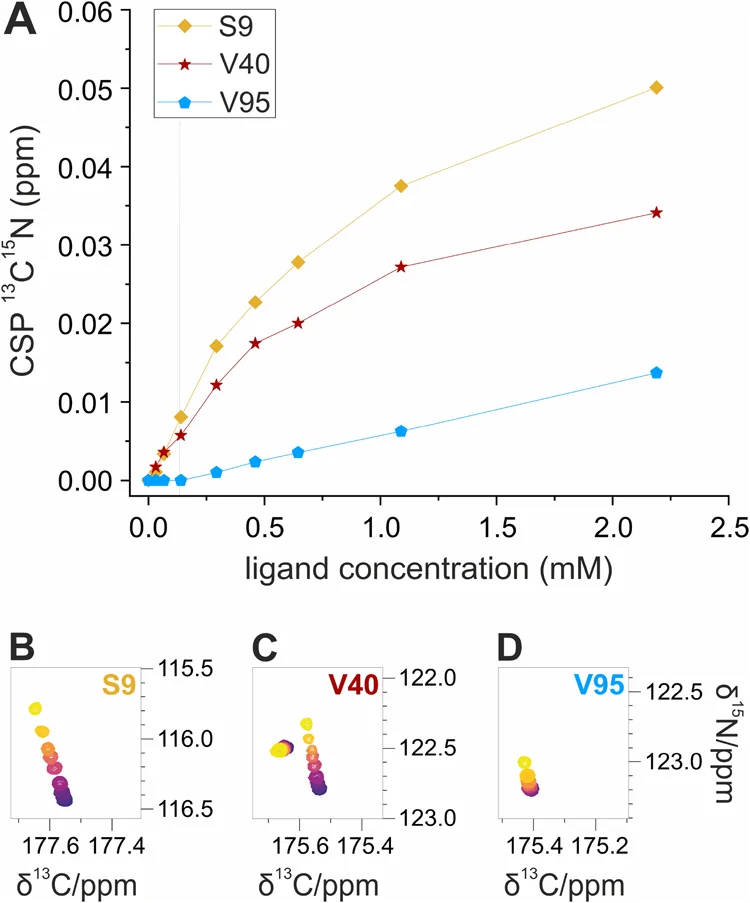
Panel A shows the chemical shift perturbations, obtained using ^13^C–^15^N CON cross peaks, for three different residues upon addition of increasing amounts of ligand CLX4S. The data for S9, V40, and V95 are shown in orange (diamonds), red (stars), and blue (pentagons), respectively. On the right, three zoomed ^13^C–^15^N CON spectral regions show the CON peaks of the ^15^N of S9 (B), V40 (C), and V95 (D) with the preceding amino acids. The spectra show the overlay of the reference spectrum (α-synuclein 310 μM, blue) with spectra acquired at α-synuclein:**CLX4S** molar ratios of 1.0:0.1 (dark violet), 1.0:0.2 (magenta), 1.0:0.4 (dark pink), 1.0:0.8 (pink), 1.0:1.2 (coral), 1.0:1.6 (orange), 1.0:2.6 (light orange), and 1.0:5.2 (yellow).

As further evidence of a complex interaction mode involving multiple sites and/or more than a single calixarene molecule, residues E20, K21, and T22 exhibit clear nonlinear chemical shift changes (Figure 4). Interestingly, the change in the direction of the chemical shift trajectory observed for residues E20, K21, and T22 occurs at an α-synuclein:**CLX4S** molar ratio of 1.0:0.8, concomitant with the onset of perturbations for the residues from V95 to K102 (Figures 4 and S1). Considering such a complex interaction picture, it is thus worth extending the focus to amino acid side chains, the primary actors promoting molecular recognition.

### Novel Tools to Study Methyl Groups in IDPs

Hydrophobic residues are often very well characterized in globular proteins and can be easily assigned through various NMR experiments.^29,30^ Thanks to the three-dimensional structure of globular proteins, the methyl group resonances are very well dispersed in 2D ^1^H–^13^C correlation experiments, and their favorable relaxation properties have been exploited to investigate very large folded proteins^70,71^ and their interactions.^72–74^

A novel variant of the 2D ^1^H–^13^C HSQC experiment optimized for IDPs and designed to resolve resonances of side chains, in particular of methyl groups, is reported in Figure S6. Experiments were acquired at ultrahigh field (1.2 GHz ^1^H Larmor frequency) to benefit from the wide dispersion of the resonances. Exploiting very high fields also allows for a better selection of subsets of spins by applying carefully designed shaped pulses.^25^ Narrowing the spectral window in the indirect dimension to the methyl region also allows us to achieve high resolution with a reduced number of increments, thereby reducing the overall experimental time. The pulse sequence was indeed modified by incorporating ^13^C band-selective shaped pulses to exclusively observe the methyl region of the spectrum (ca. 12 ppm in the ^13^C dimension centered at 17 ppm). To remove effects of homonuclear ^13^C–^13^C scalar couplings, the experiment incorporates a constant-time ^13^C evolution period. This improved experimental variant (2D ^1^H–^13^C methyl-HSQC) allowed us to acquire at high field highly resolved 2D NMR spectra focusing on the methyl region (Figure 6).

**Figure 6:**
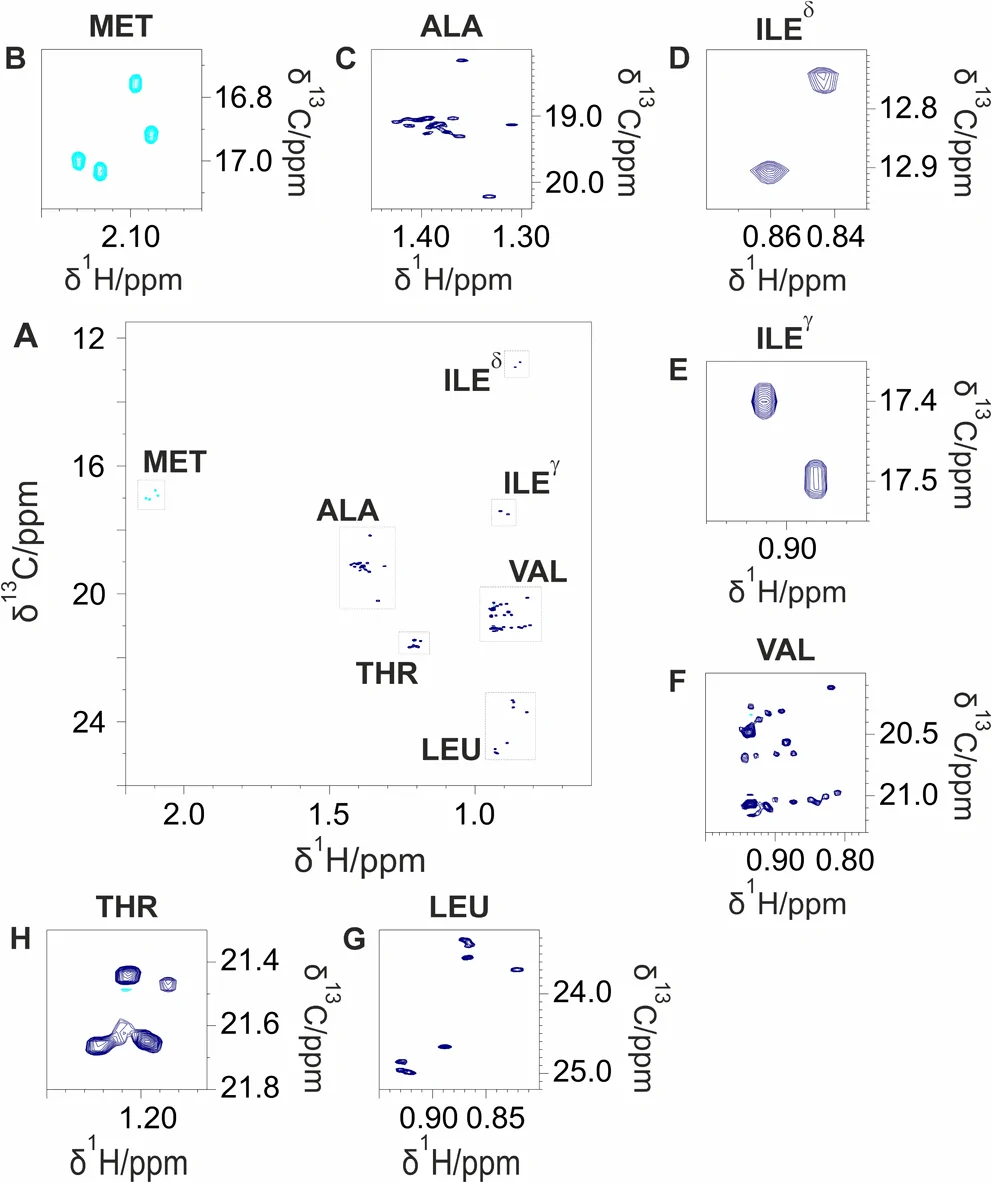
2D ^1^H–^13^C methyl-HSQC spectrum (A) acquired on a 310 μM α-synuclein sample. At first sight, the spectrum looks very crowded; however, a zoom in the different regions (panels B, C, D, E, F, G, and H) displays the excellent resolution that can be achieved through the modified pulse sequence. The spectrum was acquired at 298 K on an instrument operating at 1200 MHz (^1^H Larmor frequency).

At first glance, the 2D ^1^H–^13^C methyl-HSQC spectrum appears to have poor resolution, with all resonances arising from amino acids of the same type clustered in specific regions (Figure 6, panel A). However, a zoom into such regions highlights that most resonances can be resolved and that a high fraction of the expected correlations can be easily counted (Figure 6, panels B–H). The 2D ^1^H–^13^C methyl-HSQC spectrum can thus be used to access information on the interaction of α-synuclein with the calixarene, provided that the chemical shift assignment of the detected resonances is available.

### Side Chain Atom-Resolved Information: Assignment of Aliphatic ^1^H–^13^C Nuclear Spins

To obtain ^1^H and ^13^C chemical shift assignments of side chains, with particular focus on methyl groups, we designed a CON-based strategy relying on complementary 3D NMR experiments in which the 2D CON common element is expanded in a third indirect dimension to determine ^1^H chemical shifts of the whole aliphatic side chain (3D HBHA­(CBCA)­CON, and 3D H­(CC)­CON),^75^ as well as ^13^C chemical shifts through a constant-time version of the 3D (H)­CCCON^76^ (3D CT (H)­CCCON). Pulse sequences are reported for convenience in Figures S7, S8, and S9. Starting from the assignment of backbone nuclear spins, a viable method consists of correlating the resonances of aliphatic protons with those of the carbonyl carbon of the same amino acid (C′_i_) and of its directly bound backbone nitrogen (N_i+1_). This strategy exploits the superior resolution provided by the CON correlation (C′_i_-N_i+1_) in ^13^C-detected NMR experiments of IDPs to retrieve information about resonances of aliphatic side chains (^13^C and ^1^H chemical shifts) while taking advantage of the relaxation properties of IDPs. Of note, in all proposed experimental variants, the evolution of the ^1^H chemical shift is performed in a semiconstant time manner. This approach allows extension of the indirect dimension without limitations while simultaneously refocusing the strong ^1^H–^13^C scalar one-bond coupling, thereby yielding spectra with high resolution. Achieving such a resolution requires sampling a large number of points in the indirect dimension(s). In 3D experiments, this would lead to prohibitively long acquisition times if performed conventionally, particularly when working at high magnetic fields. To overcome this limitation, Non-Uniform Sampling (NUS) schemes can be employed.^77–82^ In our case, a reduction of the total acquisition time by a factor of 4.5 was achieved, acquiring 22% of the total number of points that would have been acquired if traditional sampling were exploited. With the actual protein concentration (310 μM), the two experiments (3D HBHA­(CBCA)­CON and the 3D H­(CC)­CON) could be acquired with optimal resolution in 40 h each, showing that they could be added to the suite of NMR experiments currently used for the investigation of IDPs, providing additional robust, accurate information. As an example of the quality of the spectra, Figure 7 reports a few strips showing the H_i_^α/β^-C′_i_-N_i+1_ correlations detected in the 3D HBHA­(CBCA)­CON with an excellent resolution. Amino acid types can easily be recognized directly from ^1^H chemical shifts, in contrast with the case of globular proteins, in which residue-type recognition is more safely achieved by inspecting ^13^C side chain resonances. Extension of the information to the whole aliphatic side chain is achieved through the 3D H­(CC)­CON experiment,^75^ as shown in the same figure.

**Figure 7:**
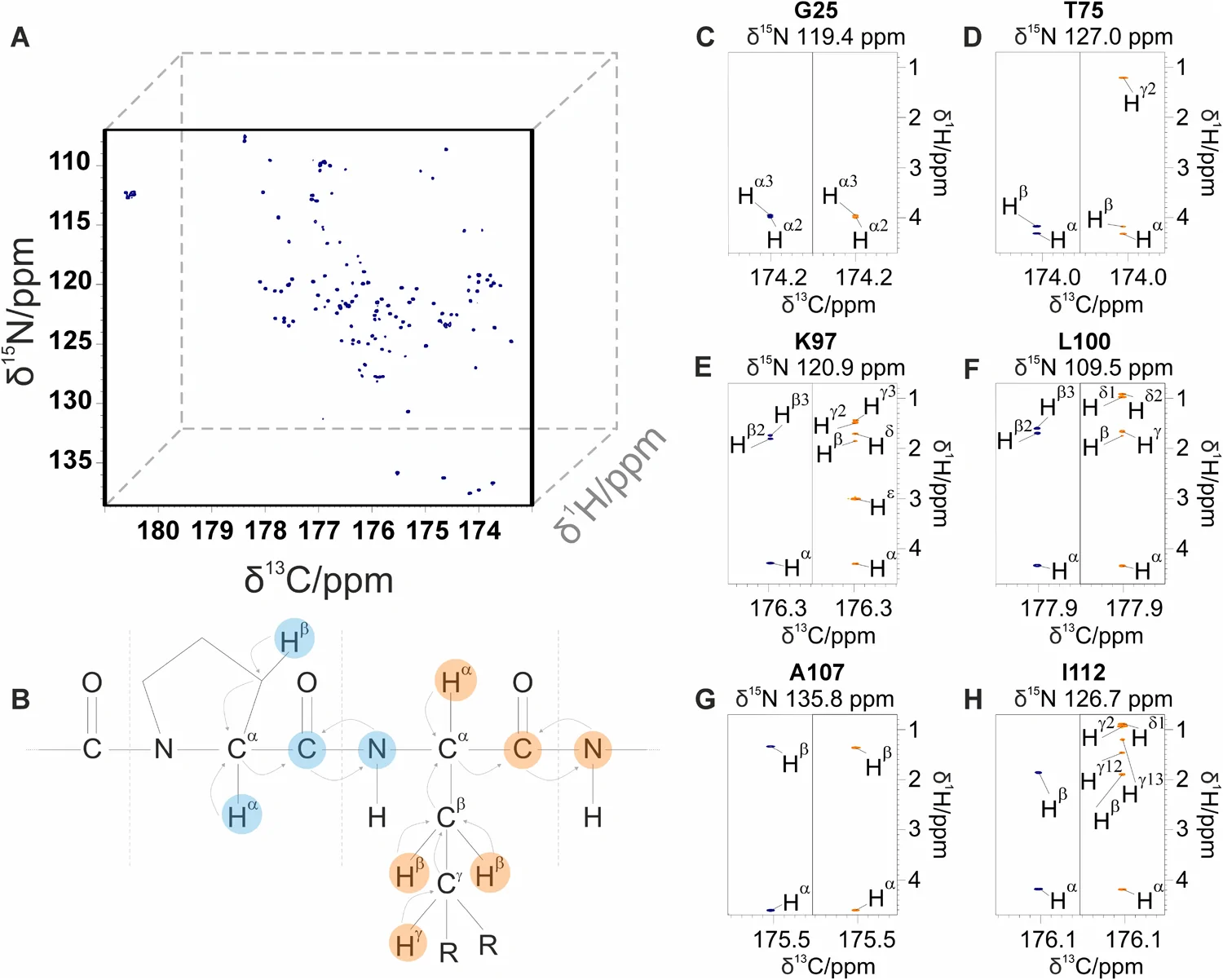
Representative results obtained through the 3D HBHA­(CBCA)­CON and the 3D H­(CC)­CON experiments acquired on a 310 μM α-synuclein sample. A schematic representation of the 3D experiment, including the ^13^C′-^15^N orthogonal plane, is shown in panel A. The observed nuclei and a schematic magnetization transfer pathway for the 3D HBHA­(CBCA)­CON and 3D H­(CC)­CON experiments are shown in panel B in blue and orange, respectively. The ^13^C′_i_-^1^H_i_^α/β^ and ^13^C′_i_-^1^H_i_^ali^ planes extracted at the corresponding ^15^N_i+1_ chemical shifts are reported in panels C–H to illustrate the achievable resolution of selected amino acids indicated at the top of each panel; the planes extracted from the 3D HBHA­(CBCA)­CON and the 3D H­(CC)­CON spectra are reported in blue and orange, respectively. The spectrum was acquired at 298 K on an instrument operating at 700 MHz (^1^H Larmor frequency).

^13^C aliphatic chemical shifts were determined through a constant-time version of the 3D (H)­CCCON^76^ (3D CT (H)­CCCON). This variant (Figure S9) improves the standard sequence for IDPs by incorporating a constant-time evolution period, during which the ^13^C aliphatic nuclear spins’ chemical shift evolves while large carbon–carbon couplings are simultaneously refocused. This results in narrower lines along the ^13^C aliphatic dimension with respect to the real-time variants, facilitating the chemical shift assignment procedure and providing accurate ^13^C chemical shift information.

In summary, this set of 3D NMR experiments benefits from the excellent resolution and completeness of the CON for IDPs and allows straightforward determination of ^1^H and ^13^C chemical shifts of aliphatic residues in one of the two indirect dimensions. Accurate determination of ^1^H and ^13^C chemical shifts is of paramount importance for the investigation of IDPs; slight changes in experimental conditions may induce chemical shift variations that are not easy to follow and challenge the identification of atom-resolved information from 2D NMR spectra. It is thus important to be able to confirm assignments in 2D fingerprint NMR spectra with the proposed approach built on the CON-based 3D NMR experiments. This experimental strategy can become generally useful in the investigation of IDPs.

Methionine residues represent a particularly challenging case and remain the only amino acid type for which a methyl group could not be assigned, exploiting the previously mentioned experiments. The presence of a sulfur in the side chain prevents efficient magnetization transfer along the side chain via ^13^C–^13^C TOCSY mixing. To overcome this limitation, a 2D ^1^H–^13^C HSQC experiment optimized for a ^3^J_CH_ correlation was employed. The resulting spectrum (Figure S10) shows two sets of correlations: one arising from the one-bond coupling (^1^J_CH_) and another arising from the three-bond coupling (^3^J_CH_). With the obtained ^13^C^γ^ assignment and repeating the experiment in the presence of the ligand, it becomes possible to firmly assign all of the four methionine methyl groups. Using the complete experimental workflow described above, all aliphatic ^1^H and ^13^C nuclear spins were assigned (100% completeness) and deposited in the BMRB under the accession code 53959.

### Monitoring Methyl Groups Involved in α-Synuclein–CLX4S Interaction

The 2D ^1^H–^13^C methyl-HSQC experiment was used to access additional information on the interaction of α-synuclein with calixarene **CLX4S**. Figure 8 illustrates, as an example, the 2D ^1^H–^13^C methyl-HSQC spectra of α-synuclein, showing the methyl resonances of leucine and methionine residues and their perturbation upon interaction with **CLX4S** (the complete set of expansions of the 2D ^1^H–^13^C methyl-HSQC spectrum is reported in Figure S11).

**Figure 8:**
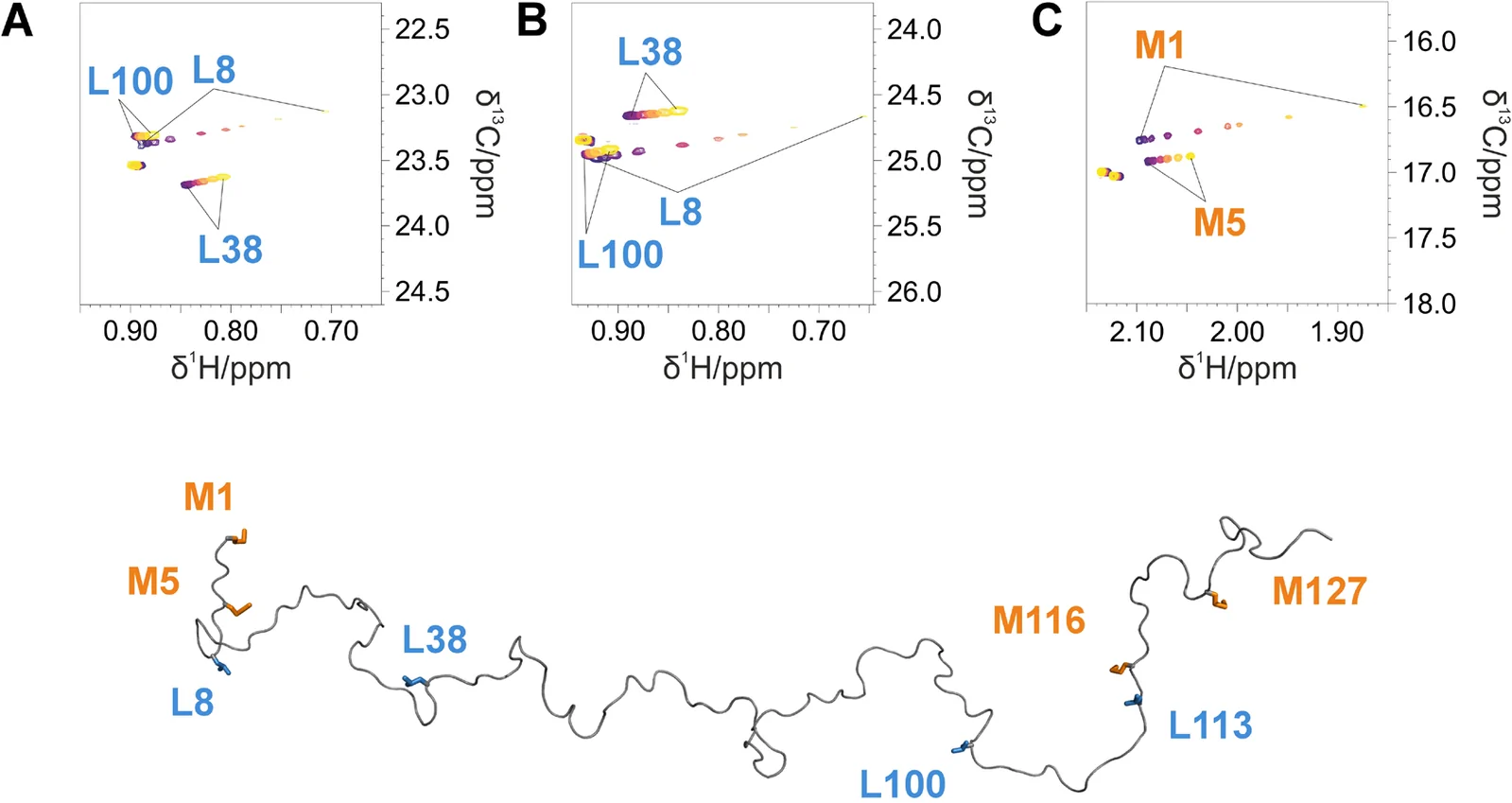
Selected regions of the 2D ^1^H–^13^C methyl-HSQC spectra acquired throughout the α-synuclein:**CLX4S** NMR titration. Panels A and B show the spectral region of leucine methyl (H^δ2^-C^δ2^ and H^δ1^-C^δ1^, respectively). Panel C shows the spectral region of methionine methyl (H^ε^-C^ε^). Each panel shows the overlay of the reference spectrum (310 μM α-synuclein sample, blue) with spectra acquired at α-synuclein:**CLX4S** molar ratios of 1.0:0.1 (dark violet), 1.0:0.2 (magenta), 1.0:0.4 (dark pink), 1.0:0.8 (pink), 1.0:1.2 (coral), 1.0:1.6 (orange), 1.0:2.6 (light orange), and 1.0:5.2 (yellow). The assignment of the most perturbed peaks is shown. The spectra were acquired at 298 K on an instrument operating at 1200 MHz (^1^H Larmor frequency). At the bottom of the figure, a possible conformer of α-synuclein is shown, with the leucine and methionine residues highlighted. The protein conformer was obtained through the combination of the Flexible Meccano^83^ and the SCWRL4 ^84^ software.

The peak experiencing the highest perturbation is the one arising from H^δ1^-C^δ1^ of leucine 8 (Figure 8, panel B). L38, located in the N-terminal region, also experiences significant perturbation. Minor, but measurable perturbations are observed for L100 (close to K96 and K97). L113, located in the C-terminal region, is instead unperturbed upon addition of **CLX4S** (Figure 8, panels A and B). Moving to the analysis of methionine methyl groups, M1 experiences a large effect, and M5 also undergoes significant perturbation upon the addition of **CLX4S** (Figure 8, panel C); this behavior is also maintained at higher ionic strength (Figure S3, panels C and D). Interestingly, the large shifts observed for methyl groups of M1 and L8 are associated with line broadening, indicative of moving into the intermediate-exchange regime on the NMR time scale. Isoleucine residues show well-resolved peaks, allowing the identification of I88 and I112; only the first one is affected by the presence of **CLX4S** (Figure S11, panels D,E). Nineteen valine residues are found in the primary structure of α-synuclein, mostly in the N-terminal and NAC regions. Among them, large perturbations are observed for V15 and V40, while the ones located in the C-terminal region are not significantly affected (Figure S11, panel F). Interestingly, the major chemical shift perturbations are upfield, consistent with CH-π interactions involving the aromatic rings of the calixarene ligand. Hydrophobic residues thus significantly contribute to the interaction, showing that charge complementarity is not the only driving force promoting binding. Although these principles are established for globular proteins and might reasonably be extended to disordered ones, to the best of our knowledge, they have not previously been explored at this level of detail in such flexible systems.

Alanine side chains are less influenced by the presence of calixarene, even though backbone analysis revealed some perturbations upon interaction (Figure S11, panel G). Threonine residues, which feature a hydroxyl group in their side chain in addition to the methyl group, are not significantly perturbed (Figure S11, panel H). Perturbations observed for backbone resonances of alanine and threonine may thus be due to indirect changes in backbone conformations rather than direct involvement in complexation of their side chains.

### Monitoring the Primary Ammonium Group of Lysine Side Chains Involved in α-Synuclein–CLX4S Interaction

Lysine residues play a key role in protein–calixarene interactions.^46,51,56^ It is thus interesting to achieve direct information on the primary ammonium group of lysine side chains through NMR, also in the challenging context of an intrinsically disordered protein such as α-synuclein. Under the experimental conditions used in this study, lysine side chains terminate in a positively charged group (N^ζ^H_3_^ζ^), preceded by a methylene group (C^ε^H_2_^ε^). The ^1^H^ε^ chemical shift can be determined through the H­(CC)­CON experiment. We collected 3D H­(CC)­CON experiments upon the addition of different amounts of **CLX4S** to α-synuclein. The results, reported in Figure 9A, indicate that ^1^H^ε^ resonances of selected lysine side chains experience an upfield shift, in agreement with previous studies performed on a dipeptide.^56^ This is consistent with the inclusion of the lysine side chain within the core of the calixarene cavity, as previously observed in the solid state for complexes between **CLX4S** and globular proteins.^46^ However, a strong modulation of the observed upfield chemical shifts is observed depending on the position of lysine residues within the α-synuclein sequence. Interestingly, lysine residues present in the N-terminal region are the most perturbed ones (K6, K10, K12, K21, K23, K32, K34, K43, K45). Modest perturbations are observed for K58, K60, K80, K96, and K97; instead, K102 is only minimally affected (Figure 9, panel B). This observation is fully consistent with the methyl group analysis, in which the residues present in the N-terminal region of α-synuclein also exhibited perturbation.

**Figure 9:**
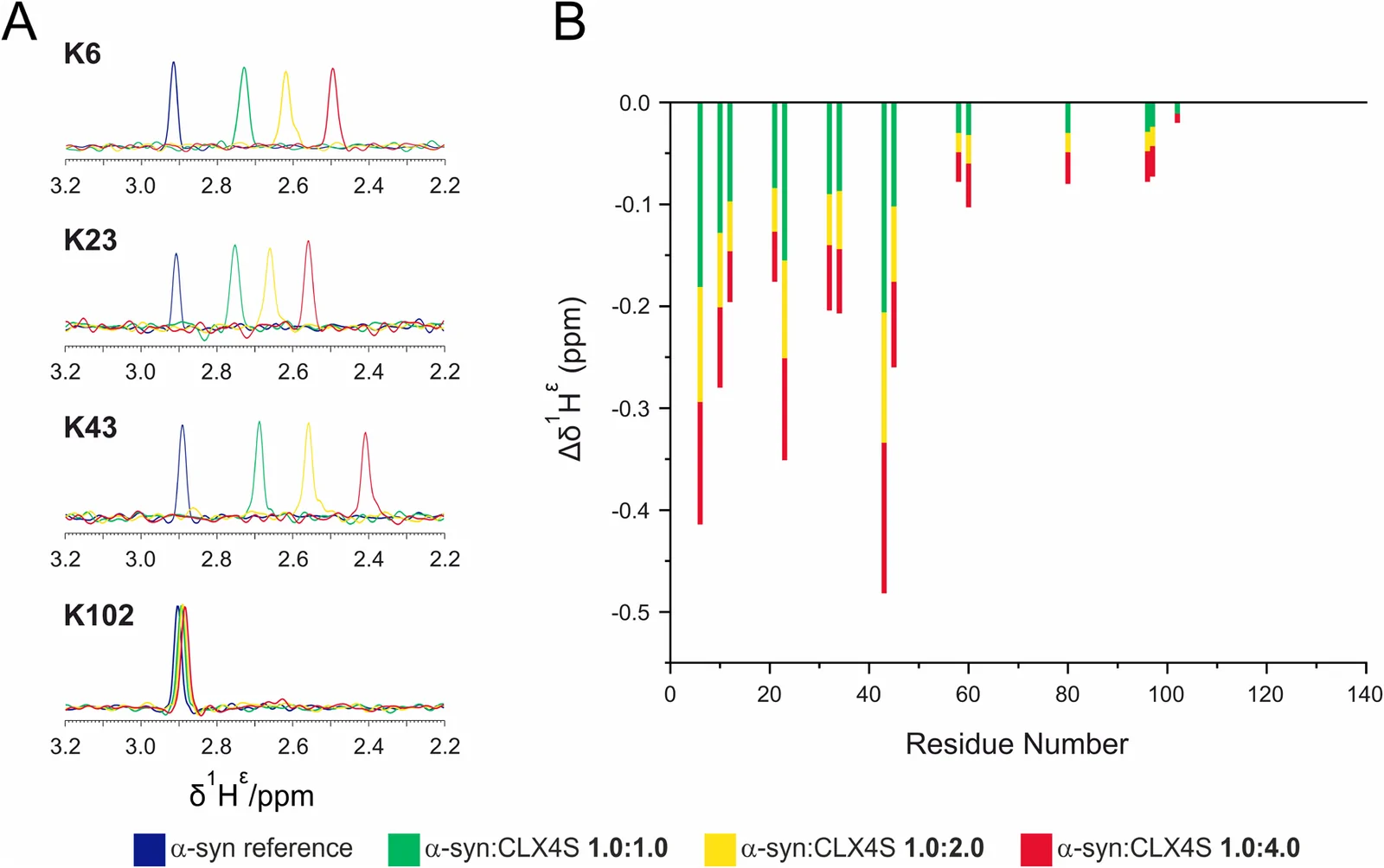
Panel A shows the ^1^H^ε^ resonances of a subset of lysine residues as observed from the 3D H­(CC)­CON experiment for the free protein (blue) and upon the addition of 1.0, 2.0, and 4.0 equiv of **CLX4S** to 1.0 equiv of protein (400 μM) in green, yellow, and red, respectively. The assignment is reported on top of each spectrum. The chemical shift variation of ^1^H^ε^ of the 15 lysine residues present in α-synuclein upon addition of 1.0, 2.0, and 4.0 equiv to 1.0 equiv of protein is reported in panel B. The spectra were acquired at 298 K on an instrument operating at 700 MHz (^1^H Larmor frequency).

Interestingly, although the two analyses are generally consistent, the residues showing the largest perturbations in their backbone resonances do not always exhibit the largest shifts in their ^1^H^ε^ resonances, as the latter are located five covalent bonds away. From the backbone point of view, K10, K12, K21, K43, K96, and K97 were found to be highly shifted, while K6, K23, K34, K45, K58, K60, K80, and K102 showed only minor perturbations. This shows the importance of novel NMR tools to access more information about IDPs’ interactions to investigate complex systems.

### Monitoring Aromatic Residues Involved in α-Synuclein–CLX4S Interaction

The calixarene ligands are also characterized by a highly aromatic component, suggesting that aromatic side chains of α-synuclein may also contribute to binding. We therefore employed a 2D ^1^H–^13^C TROSY HSQC experiment including a constant-time period evolution (2D ^1^H–^13^C CT-TROSY HSQC), previously used to investigate the interplay of different ligands with α-synuclein,^28^ to probe the involvement of aromatic residues.

Upon addition of **CLX4S** to α-synuclein, Y39 experiences an upfield shift, as shown in Figure S12. Interestingly, Y39 is close to other residues whose side chains are highly perturbed (L38, V40). A behavior like the one described for H^ε^C^ε^ of Y39 can also be observed for the signals of the two phenylalanine residues (F4 and F94), also close to other perturbed residues, as shown in Figure S13. This complex pattern reveals details on the interaction mechanism. As the ligand concentration increases, specific upfield shifts emerge for F4, Y39, and F94. These shifts may arise from π–π stacking or C–H··π interactions, as previously reported for other aromatic compounds shown to interact with tyrosine residues of α-synuclein.^28,65^

## Discussion

### Flexible Protein Regions Interact with Multivalent, Rigid Scaffolds

The rational design of ligands for IDPs is by no means a trivial task.^85^ Current approaches in drug discovery target protein binding pockets that, by definition, are not present in highly flexible, disordered proteins. The example of differently functionalized calixarene molecules highlights, in a very clean and clear way, some general features that can be sought when searching for tailored ligands targeting specific regions of IDPs. From the chemical point of view, the proposed ligands are characterized by a common aromatic scaffold, which defines a rather rigid hydrophobic cavity, and a variable upper rim. Both these elements of the molecule are necessary for efficient interaction. The major driving forces addressing the investigated ligands toward selected regions of α-synuclein are long-range charge–charge interactions. The functions at the calixarene upper rim guide the molecule to different parts of the protein: the cationic molecules preferentially interact with the α-synuclein’s region enriched in negatively charged residues (C-terminal region), while the anionic calixarene is guided to the positively charged region (N-terminal region). The multivalent and relatively rigid nature of calixarene compounds constitutes an important feature to enable the formation of a number of local interactions with different residues in the primary structure, still in a dynamic mode.^65,67,86,87^ Indeed, the high flexibility and disorder of the protein enable continuous formation-disruption of multiple local interactions that contribute to increasing the affinity for the ligand.

Electrostatics is not the only driving force promoting the interaction of calixarene ligands with α-synuclein. Even in a highly disordered state, solvent-exposed positively and negatively charged residues do not equally sense the presence of ligands but rather do so in a context-dependent manner, in other words, depending on the protein region in which they are embedded. Figure 10 shows, in a pictorial way, the interaction hotspots for the three different calixarenes.

**Figure 10:**
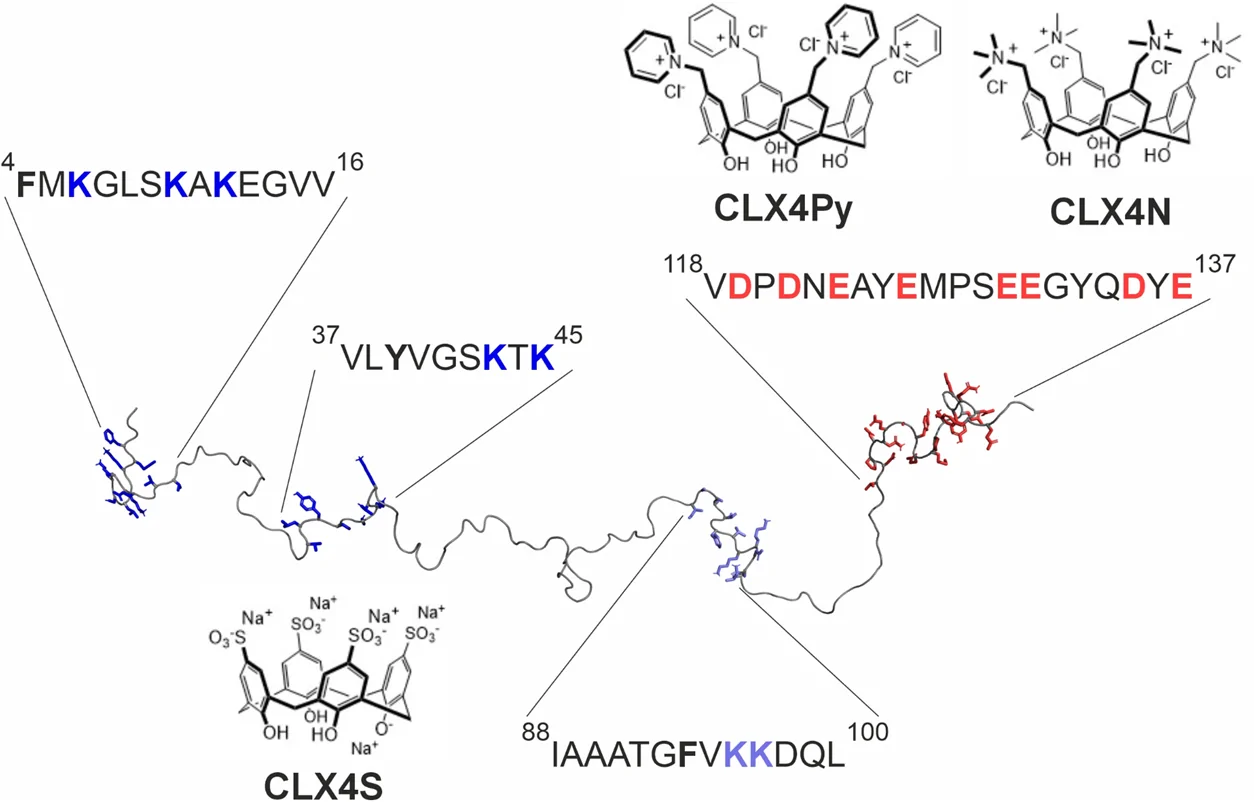
Three proposed ligands and their interaction regions as derived from NMR data. One of the possible conformers of α-synuclein is reported, highlighting the interacting regions. **CLX4S** and its primary binding sites are reported in blue. The secondary binding region of **CLX4S** is reported in light blue. **CLX4Py** and **CLX4N** and their interaction sites are reported in red. The protein conformer was obtained through the combination of the Flexible Meccano^83^ and the SCWRL4 ^84^ software.

### Framing Our Results on the Protein Sequence

Focusing in more detail on the **CLX4S**/α-synuclein complexation, multiple interaction sites with different affinities can be identified that are progressively populated upon the addition of increasing amounts of ligand. Two major binding regions can be observed involving residues F4–V16 and V37–K45. An additional region, spanning residues I88-L100 and including two lysine residues at positions 96 and 97, becomes significantly perturbed upon ligand addition only at higher ligand concentrations, indicative of a lower-affinity binding site. The presence of lysine residues in all of the protein regions significantly affected by the presence of **CLX4S** is in agreement with the well-known ability of this anionic macrocycle to bind the side chain of this amino acid in globular proteins.^88^ ,^89^ ,^46^ In case of IDPs, thanks to the new NMR experiments proposed, we have observed a similar effect by looking at the ^1^H^ε^ chemical shift perturbation upon addition of the ligand to the protein, suggesting a similar behavior.

Inspection of methyl groups through the 2D ^1^H–^13^C methyl-HSQC experiment reveals interesting insights. Side chain signals belonging to the same residue types are not uniformly affected, and the perturbation is strongly influenced by the position of the residues along the sequence, with differences observed between the N- and C-terminal regions as exemplified by the case of leucine and methionine residues: two leucine residues are found in the primary binding sites (L8 and L38) and are highly perturbed, one is located in the secondary binding site (L100) and experiences only a mild effect, and the fourth one, located in the C-terminal region, is unaffected (L113). Similarly, only two of the four methionine residues (M1 and M5) experience significant chemical-shift perturbations.

Most importantly, the upfield chemical shift perturbations observed for methyl resonances of leucine and methionine side chains, as well as of ^1^H^ε^ of lysine residues, with an aromatic ligand such as **CLX4S**, provide a clear signature of CH-π interactions (methyl groups located along the perpendicular direction to the aromatic ring planes).^74,90^ This represents a short-range directional interaction, highly complementary to charge–charge electrostatic interactions, which are isotropic and sensed at long distances. In the case of methionine, CH-π interactions were directly observed in the X-ray structure of the dynamic N-terminus of a globular protein complexed with **CLX4S**, where the amino acid side chain was encapsulated within the macrocyclic cavity.^52^ This suggests that the observed upfield chemical shift perturbations may likewise arise from encapsulation of these residues in the present system, and that the same mode of binding might also be extended to leucine residues. This would provide an alternative mode of protein binding that the ligand may exploit in addition to typical lysine recognition. On the other hand, a similar upfield shift could arise from interaction of a side-chain methyl group with the external face of a calixarene bound to another residue, a situation that we cannot rule out at this stage.

Therefore, the present results show a clear example of how charge–charge and hydrophobic effects contribute to α-synuclein/calixarene recognition. Another important element governing protein recognition seems to be the structure complementarity between the calixarene ligands and the target amino acid side chain. Indeed, comparing the same binding region, either in F4–V16, V37–K45, or I88-L100, the amino acids with longer side chains are generally found to be more perturbed with respect to the ones with shorter side chains. This trend is particularly evident for aliphatic residues, with leucine being among the most perturbed residues (Figure 8) and alanine residues the least perturbed ones, even when present in the highly perturbed protein regions (Figure S11). This could be due to the bulkiness of the calixarene macrocycle, which more easily accesses longer and solvent-exposed side chains rather than shorter ones, which are closer to the backbone and thus more hindered. The same hypothesis may apply to the two methionine residues experiencing very large CSPs, whose relatively long and linear side chains could be particularly accessible to the tested calixarene. By contrast, valine residues’ side chains are more perturbed than threonine ones, although sharing the same side chain length as determined by the number of aliphatic carbons. However, the hydrophilic character of threonine deriving from the hydroxyl group probably makes this residue less suited to engage the lipophilic scaffold of **CLX4S**.

Aromatic residues are also found to play a role in the interaction. The resonances of Y39 (Figure S12), F4, and F94 (Figure S13) are indeed perturbed upon addition of the molecule in a way that can be reconciled with the presence of π–π stacking or CH–π interactions (Figures S12 and S14). These residues are found in regions showing higher affinity for **CLX4S** and show a different behavior with respect to the tyrosine residues found in the C-terminal region (Y125, Y133, Y136).

Taken together, these results reveal common features of the most affected regions in α-synuclein upon interaction with **CLX4S**, namely, the presence of charged residues in short linear motifs in which auxiliary aliphatic and aromatic residues are present.

High-affinity sites comprise a KXK motif (as in ^10^KAK^12^ and ^43^KTK^45^) at variance with the lower-affinity one, which features a KK motif. The KXK motifs might be more effective in binding to CLX4S also due to the presence of a residue in between two lysine amino acids, which, in elongated protein conformations, are expected to orient lysine side chains in the same direction, facilitating in this way the electrostatic attraction of the bulky ligand. However, other KXK motifs are present in the primary structure of the protein (^32^KTK^34^, ^58^KTK^60^), which only experience minimal chemical shift perturbations upon interaction with the macrocycle. While in globular proteins a similar selectivity among different lysine residues could be explained by their different degree of accessibility, a similar argument does not hold for an intrinsically disordered protein such as α-synuclein, in which lysine residues are largely solvent exposed. Therefore, other features must be responsible for the higher affinity of **CLX4S** for the three identified regions (F4–V16, V37–K45, and I88-L100) with respect to the remaining part of the protein sequence. The available data on methyl groups, lysine side chains, and aromatic residues, which report about direct contacts with the ligand, provide useful information to answer this question (Figure 8).

### Framing our Results in the Scientific Literature

For many years, research has focused on the investigation of the three-dimensional structures of fibrils. Recent progress revealed how different fibril morphologies can be correlated to distinct neurodegenerative diseases.^91^ However, once fibrils are formed, it is not easy to identify strategies for their reversal toward the native state due to their remarkable thermodynamic stability. Moreover, the disaggregation of fibrils can produce synuclein oligomers that are by now recognized as the most toxic species responsible for neurodegeneration.^92^ It is thus important to shift the focus to the native intrinsically disordered state of α-synuclein and to approaches that help maintain it in its monomeric state. While we have made great progress on the investigation of IDPs,^93–96^ we still need to learn how they interact with potential partners in the cell able to modulate their energy landscapes (metal ions, small molecules, proteins, nucleic acids, lipids, etc.), how they are affected by post-translational modifications (PTMs), and how they can be targeted by external ligands.

In this frame, calixarene ligands have been recently proposed as valuable candidates to target α-synuclein in its disordered state.^55,43,56^ Recent work showed through multiple techniques that, among a panel of different calix[4]­arene ligands, the tetraphosphonato variant behaves as a potent inhibitor of α-synuclein aggregation in a validated yeast model of PD based on α-synuclein overexpression.^56^ Here, we wanted to understand the driving forces underlying calixarene-α-synuclein interactions as initial steps toward rational design of ligands to target IDPs. The clear results obtained also allow us to inspect recent examples of α-synuclein-small molecule interactions^28,65,67^ in a more general context. Future studies will determine whether the main driving forces identified for the calixarene ligands investigated here are generally applicable to the targeting of flexible protein regions. Calixarene molecules also constitute an interesting example as they represent a relatively rigid scaffold which also features a “cavity” in which specific amino acid side chains can be hosted. Designing “cavities” in which short linear motifs of IDPs can adapt has recently been proposed as an extension of de novo protein design.^97^ Calixarenes could thus also be seen as ligands that provide the initial template of a cavity, facilitating interactions also with highly flexible protein regions.

## Conclusions

A detailed investigation of the recognition process between α-synuclein and calix[4]­arene-based ligands has been performed, elucidating the different interactions occurring and the origin of the observed selectivity alongside the protein sequence. The high level of insight was possible thanks to extensive use of high-field NMR techniques. The novel NMR experiments proposed are simple, sensitive, and provide excellent resolution thanks to the favorable relaxation properties of IDPs; acquisition of such experiments at high magnetic fields provides a further boost in resolution. They can be used for any IDP investigation, and the information obtained from ^1^H and ^13^C chemical shifts of amino acid side chains opens new possibilities. One of them consists of the study of the role of side chains in interactions, as demonstrated in the present case. In perspective, the availability of ^1^H chemical shifts beyond the backbone can prove very useful to determine additional observables such as relaxation rates, NOEs (Nuclear Overhauser Effect), or PREs (Paramagnetic Relaxation Enhancement) that can be used to characterize IDPs’ conformational ensembles. Populating the BMRB database with information about side chains would greatly improve our knowledge of how the properties of IDPs are modulated by external conditions and by interactions with different types of ligands (metal ions, small molecules, proteins, and nucleic acids, etc.). This would also represent a significant step toward providing the experimental data for the development of AI-based tools for the assignment and characterization of IDPs.

## Experimental Section

### Synthesis of 5,11,17,23-Tetrakis­[(pyridinium)­methyl]-25,26,27,28-tetrahydroxycalix[4]­arene Tetrachloride (CLX4Py)

A suspension of tetrachloromethylcalix[4]­arene^63^ (0.100 g, 0.16 mmol) in pyridine (1 mL) was heated at 80 °C for 6 h. After that, the solvent was evaporated under reduced pressure. The residue was triturated with diethyl ether, and the product was collected by filtration as a pale-yellow solid in 68% yield (0.102 g, 0.11 mmol). ^1^H NMR (400 MHz, D_2_O) δ (ppm): 8.64 (d, *J* = 6.1 Hz, 8H, pyridinium *H*_*ortho*_), 8.39 (t, *J* = 7.8 Hz, 4H, pyridinium *H*_*para*_), 8.20 (bs, 4H, Ar–O*H*), 7.87 (t, *J* = 7.0 Hz, 8H, pyridinium *H*_*meta*_), 7.09 (s, 8H, Ar-*H*), 5.42 (s, 8H, Ar–C*H*_2_-pyridinium), 3.78 (s, 8H, Ar–C*H*_2_-Ar), Figure S15. ^13^C NMR (101 MHz, D_2_O) δ (ppm): 154.8 (Ar*C*–OH), 145.5 (pyridinium *C-*H_*para*_), 143.4 (pyridinium *C-*H_*ortho*_), 131.3 (Ar*C*-CH_2_–Ar), 129.8 (Ar*C*-H), 128.0 (pyridinium *C-*H_*meta*_), 123.4 (Ar*C*-CH_2_–pyridinium), 64.1 (Ar-*C*H_2_-pyridinium), 31.6 (Ar-*C*H_2_–Ar), Figure S16. ESI-MS: *m*/*z* calc 934.78, obs: 273.12 (100%) [M-pyridine, mono-quinone methide]^3+^; 473.14 (93%) [M-4 pyridines, tetra-quinone methide]^+^.

### Protein Production

The ^15^N- and ^13^C–^15^N-labeled α-synuclein samples were prepared as previously described in the literature.^98^

### NMR Samples

For NMR titrations of ^15^N α-synuclein with **CLX4Py**, **CLX4N**, and **CLX4S**, experiments were acquired using a 5 mm tube containing 500 μL of 200 μM protein in TRIS buffer (25 mM TRIS, 25 mM KCl at pH 7.2), with 5% D_2_O added for the lock. In the case of **CLX4Py** and **CLX4N** the stock solutions were prepared at a final concentration of 1.0 mM in the same buffer, while for **CLX4S**, the stock solution was prepared at a final concentration of 20 mM in the same buffer. The titration points were selected based on the protein’s spectral response and correspond to the following molar ratios:

^15^N α-synuclein:CLX4Py. 1.0:0.0, 1.0:0.1, 1.0:0.2, 1.0:0.3, 1.0:0.4, 1.0:0.6, 1.0:0.8.

^15^N α-synuclein:CLX4N. 1.0:0.0, 1.0:0.2, 1.0:0.4, 1.0:0.6, 1.0:0.7, 1.0:0.8, 1.0:0.9, 1.0:1.0, 1.0:1.6.

^15^N α-synuclein:CLX4S. 1.0:0, 1.0:0.2, 1.0:0.4, 1.0:0.8, 1.0:1.6, 1.0:3.2, 1.0:6.4.

For the NMR titration of ^13^C–^15^N α-synuclein with **CLX4S**, experiments were acquired using a 5 mm tube containing 500 μL of 310 μM protein in TRIS buffer, with 5% D_2_O added for the lock. A second NMR titration was performed using a 5 mm tube containing 500 μL of 400 μM protein in TRIS buffer, with 5% D_2_O added for the lock. A third NMR titration at high ionic strength (100 mM KCl) was acquired using a 5 mm tube containing 500 μL of 310 μM protein in TRIS buffer, with 5% D_2_O added for the lock. A stock solution of **CLX4S** was prepared with a final concentration of 20 mM in the same NMR buffer. Aliquots of the **CLX4S** stock solution were added to reach the following molar equivalents of α-synuclein to calixarene ligands: 1.0:0.0, 1.0:0.1, 1.0:0.2, 1.0:0.4, 1.0:0.8, 1.0:1.2, 1.0:1.6, 1.0:2.6, and 1.0:5.2. In the second NMR titration, aliquots of the **CLX4S** stock solution were added to reach the following molar equivalents of α-synuclein to calixarene ligands: 1.0:0.0, 1.0:1.0, 1.0:2.0, and 1.0:4.0. In the third NMR titration, aliquots of the **CLX4S** stock solution were added to reach the following molar equivalents of α-synuclein to calixarene ligands: 1.0:0.0, 1.0:0.4, 1.0:0.8, 1.0:1.2, 1.0:1.6, 1.0:2.6, and 1.0:5.2.

### NMR Experiments

The following spectrometers were used:

- A Bruker AVANCE NEO spectrometer operating at 700.13 MHz ^1^H, 176.03 MHz ^13^C, and 70.93 MHz ^15^N frequencies equipped with a cryogenically cooled probe head optimized for ^13^C-direct detection (CP-TXO), namely, 700.
- A Bruker AVANCE NEO spectrometer operating at 950.20 MHz ^1^H, 238.93 MHz ^13^C, and 96.28 MHz ^15^N frequencies, equipped with a cryogenically cooled probe head optimized for ^1^H-direct detection (TCI), namely, 950.
- A Bruker AVANCE NEO spectrometer operating at 899.64 MHz ^1^H, 226.21 MHz ^13^C, and 91.16 MHz ^15^N frequencies equipped with a cryogenically cooled probe head optimized for ^1^H-direct detection (TCI), namely, 900.
- A Bruker AVANCE NEO spectrometer operating at 1200.73 MHz ^1^H, 301.92 MHz ^13^C, and 121.67 MHz ^15^N equipped with a cryogenically cooled probe head optimized for ^13^C-direct detection (CP-TXO), namely, 1200.

To follow the titrations of ^15^N-labeled α-synuclein with **CLX4Py**, **CLX4N**, and **CLX4S**, a series of 2D ^1^H–^15^N BEST TROSY experiments were recorded for **CLX4Py** and **CLX4S** at 950, while for **CLX4N**, experiments were acquired at 900. All of the experiments were conducted at a temperature of 298 K. The acquisition parameters of NMR experiments are reported in Table 1.

**Table 1:** NMR Acquisition Parameters Used for all 2D ^1^H–^15^N BEST TROSY Experiments on **CLX4Py**, **CLX4N**, and **CLX4S**

compound	data points		spectral width (Hz)		NS^a^	recovery delay (s)^b^	Frequency (MHz)
	F2	F1	F2	F1			
CLX4Py	4096	512	13888	2407	8	0.34	950
CLX4N	4096	512	8198	2369	4	0.50	900
CLX4S	4096	512	13888	2407	8	0.34	950

a Number of acquired scans.

b Recovery delay (d1) plus acquisition time.

The titration of ^13^C–^15^N-labeled α-synuclein with **CLX4S** was performed at 1200. The experiments were conducted at a temperature of 298 K. The acquisition parameters of NMR experiments are reported in Table 2.

**Table 2:** Acquisition Parameters Used for the 2D NMR Experiments on α-synuclein:**CLX4S**

experiments	data points		spectral width (Hz)		NS^a^	recovery delay (s)^b^
	F2	F1	F2	F1		
BEST TROSY	8192	512	19230	3533	4	0.41
CON	2048	650	7462	4015	4	2.13
CACO	2048	628	7462	11495	4	2.13
CBCACO	2048	826	7462	19628	4	1.13
HC TROSY (aro)	8192	532	16666	7936	4	1.54
HC HSQC (met)	4096	436	19230	8474	4	1.40

a Number of acquired scans.

b Recovery delay (d1) plus acquisition time.

The titration of ^13^C–^15^N-labeled α-synuclein with **CLX4S** at high ionic strength was performed at 950. The experiments were conducted at a temperature of 298 K. The acquisition parameters of NMR experiments are reported in Table 3.

**Table 3:** Acquisition Parameters Used for the 2D NMR Experiments on α-Synuclein:**CLX4S** at High Ionic Strength

experiments	data points		spectral width (Hz)		NS^a^	recovery delay (s)^b^
	F2	F1	F2	F1		
BEST TROSY	4096	866	13888	2888	4	0.41
CON	2048	514	8196	3177	4	2.13
CACO	2048	496	8196	9082	4	2.13
HC TROSY (aro)	6184	420	13157	6281	4	1.54
HC HSQC (met)	4096	344	19230	6711	4	1.40

a Number of acquired scans.

b Recovery delay (d1) plus acquisition time.

The NMR experiments used to obtain the sequence-specific assignment on side chains were acquired at 700. In the 3D HBHA­(CBCA)­CON, 3D H­(CC)­CON, and 3D (H)­CCCON, the ^1^H carrier was placed at 2.5 ppm. The center of ^13^C band-selective pulses was set at 176.7, 45.7, and 55.9 ppm for C′, C^ali^, and C^α^ regions. ^15^N pulses were given at 123.0 ppm. Q5 and Q3 shapes^99^ of durations of 300 and 231 μs were used for ^13^C band-selective π/2 and π flip angle pulses, respectively, except for the adiabatic π pulse to invert both C′ and C^α^ (smoothed Chirp^100^ 500 μs, 20% smoothing, 80 kHz sweep width, 11.2 kHz field strength). Composite pulse decoupling was applied on ^1^H (Garp-4 ^101^) with an RF field strength of 2.5 kHz. ^15^N decoupling was achieved with an RF field strength of 1.0 kHz through the garp4 ^101^ composite pulse decoupling scheme. ^13^C homonuclear decoupling was achieved through IPAP virtual decoupling.^102^

In the 3D H­(CC)­CON and 3D (H)­CCON experiments, a TOCSY mixing block was applied for 28 ms with an RF field strength of 8.3 kHz.

The remaining experimental parameters (type of experiment, spectral width, acquired data points, acquisition times, number of scans, interscan delays, and NUS percentage) are reported in Table 4. The acquisition parameters reported in Table 4 were used to obtain the sequence-specific assignment C^α/β^, C^ali^, H^α/β^, and H^ali^, exploiting the pulse sequences reported in Figures S7–S9.

**Table 4:** NMR Acquisition Parameters Used for Obtaining the Sequence-Specific Assignment of C^α/β^, C^ali^, H^α/β^, and H^ali^ on α-synuclein

experiment	dimension of acquired data and total evolution			spectral width (ppm)			NS^a^	recovery delay (s)^b^	NUS amount (%)	frequency (^1^H MHz)
	t_1_	t_2_	t_3_	F1	F2	F3				
HBHA(CBCA)CON	256 (^1^H) 45 ms	192 (^15^N) 39 ms	2048 (^13^C) 143 ms	4	34	41	4	1.3	22	700
H(CC)CON	256 (^1^H) 36 ms	192 (^15^N) 39 ms	2048 (^13^C) 143 ms	5	34	41	4	1.3	22	700
CT (H)CCCON	256 (^13^C) 11 ms	192 (^15^N) 39 ms	2048 (^13^C) 143 ms	66	34	41	4	1.3	22	700

a Number of acquired scans.

b Recovery delay (d1) plus acquisition time.

The 3D H­(CC)­CON experiment was repeated using the same parameters, also to follow the interaction of α-synuclein with **CLX4S** molar ratios of 1.0:0.0, 1.0:1.0, 1.0:2.0, and 1.0:4.0.

### NMR Spectral Analysis

All acquired NMR spectra were processed using Bruker TopSpin 4.5.0, and chemical shifts were referenced to the DSS (sodium 2,2-dimethyl-2-silapentane-5-sulfonate) standard.^103^ Spectral analysis was carried out using CARA^104^ to perform chemical shift assignment and utilizing its tool NEASY for the chemical shift perturbation analysis. For each titration point, peak positions were manually adjusted to accurately track chemical shift variations upon ligand addition. This procedure enabled the calculation of chemical shift perturbation (CSP) values, which were subsequently analyzed.^105^ CSP values in ^1^H–^15^N spectra were determined according to the following equation:

$$CSP=\sqrt{\frac{1}{2}\left[{\left(\delta_{H}\right)}^{2}+{\left(K1*\delta_{N}\right)}^{2}\right]}$$

To determine the CSP values in the CON spectra, the following formula was instead employed:

$$CSP=\sqrt{\frac{1}{2}\left[{\left(K2\times \delta_{\mathrm{CO}}\right)}^{2}+{\left(K1\times \delta_{N}\right)}^{2}\right]}$$

where δ_H_, δ_N_, and δ_CO_ are the differences in the chemical shift value between the peak position measured in the presence of the ligand and the initial chemical shift value for the same peak in the proton, nitrogen, and carbonyl dimensions, respectively, and *K*1 and *K*2 are constants; for this work, we used *K*1 = 0.1 and *K*2 = 0.3.

The dissociation constant (*K*_d_) for the interaction between α-synuclein and **CLX4S** was determined by measuring the CSP for each peak in a series of 2D CON spectra recorded at increasing concentrations of the ligand. The data were fitted using the following equation:^105^

$$CSP=\frac{P+L+K-\sqrt{{\left(P+L+K\right)}^{2}-4\mathrm{PL}}}{2P}\cdot M$$

with CSP previously defined, *P* is the total protein concentration, *L* is the **CLX4S** concentration at different titration points, *M* represents the maximum expected CSP value upon the complete formation of the bound state, and *K* is the dissociation constant (*K*_d_).

## Supplementary Material



## Notes

CSP of α-synuclein as obtained from ^13^C–^15^N and ^1^H–^15^N experiments (Figure S1); CON full spectrum of α-synuclein upon addition of **CLX4S** (Figure S2); selected regions of the ^13^C–^15^N CON and ^1^H–^13^C methyl-HSQC spectra of α-synuclein upon addition of **CLX4S** at low and high ionic strength concentration (Figure S3); selected regions of the CACO spectrum of α-synuclein upon addition of **CLX4S** (Figure S4); fit of CSP against ligand concentration for S9 and V40 (Figure S5); scheme reporting the pulse sequence for ^1^H–^13^C methyl-HSQC (Figure S6); scheme reporting the pulse sequence for HBHA­(CBCA)­CON (Figure S7); scheme reporting the pulse sequence for H­(CC)­CON (Figure S8); scheme reporting the pulse sequence for (H)­CCCON (Figure S9); spectrum of long-range ^1^H–^13^C HSQC on methionine signals (Figure S10); zoom on different regions of the 2D ^1^H–^13^C methyl-HSQC spectrum of α-synuclein upon addition of **CLX4S** (Figure S11); zoom on the tyrosine region of the ^1^H–^13^C TROSY HSQC spectrum of α-synuclein upon addition of **CLX4S** (Figure S12); zoom on the phenylalanine region of ^1^H–^13^C TROSY HSQC spectrum of α-synuclein upon addition of **CLX4S** (Figure S13); pictorial description of possible interaction causing upfield shift in the NMR signals (Figure S14); ^1^H NMR spectrum of **CLX4Py** (Figure S15); ^13^C NMR spectrum of **CLX4Py** (Figure S16) (PDF)
