# Supplementary material for: Toward the Rational Design of Tailored Ligands for Intrinsically Disordered Proteins through NMR
Source: J Am Chem Soc. 2026 Oct 22;148(43):47337–53. doi: 10.1021/jacs.6c16561 (PMC13741821; doi:10.1021/jacs.6c16561)
Supplement: Supplementary file 1 [file ja6c16561_si_001.pdf]

Towards the rational design of tailored ligands for intrinsically disordered proteins through NMR

Cristina Ciabini, [# a, b] *Filippo Turchi,* [# a, b] *Marco Schiavina,* * [a, b, c] *Tommaso Materassi,* [a, b] *Filippo Bonini,* [a, b] *Maria Cremonini,* [d] *Francesca Clemente,* [a] *Francesca Cardona,* [a] *Stefano Volpi,* [d] *Laura Baldini,* [d] *Francesco Sansone,* * [d] *Roberta Pierattelli,* * [a, b, c] *Isabella C. Felli** [a, b, c]

[a] Department of Chemistry "Ugo Schiff" (DICUS), University of Florence, 50019 Sesto Fiorentino, FI, Italy;

[b] Magnetic Resonance Center (CERM), University of Florence, 50019 Sesto Fiorentino, FI, Italy;

[c] Consorzio Interuniversitario Risonanze Magnetiche Metallo Proteine (CIRMMP), 50019 Sesto Fiorentino, Italy;

[d] Department of Chemistry, Life Sciences and Environmental Sustainability, University of Parma, 43124 Parma, Italy;

*Corresponding authors: Marco Schiavina (marco.schiavina@unifi.it), Francesco Sansone (francesco.sansone@unipr.it), Roberta Pierattelli (reoberta.pierattelli@unifi.it) and Isabella C. Felli (isabellacaterina.felli@unifi.it).

[#] Cristina Ciabini and Filippo Turchi contributed equally to this paper.

Supporting Information

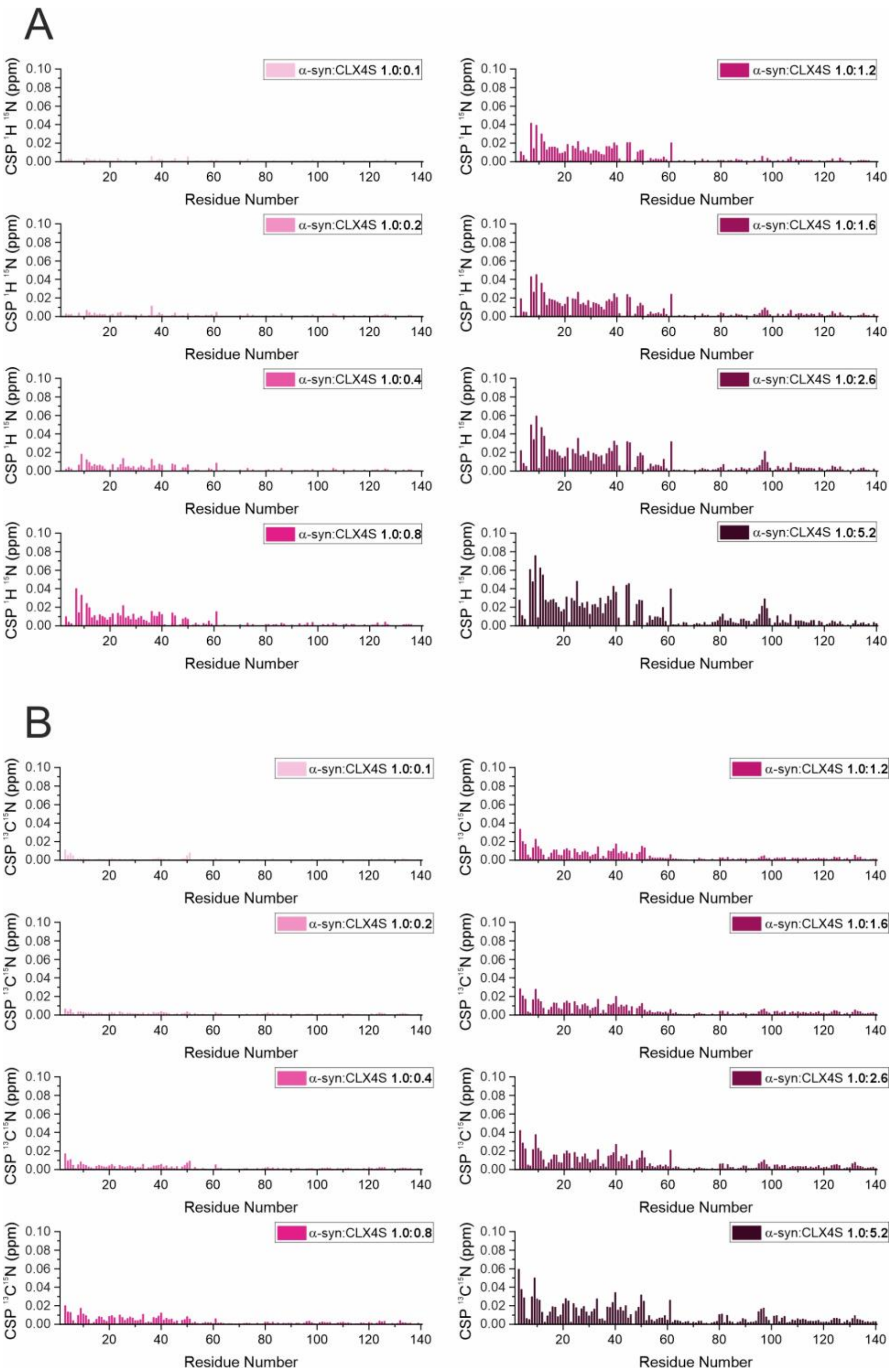

Figure S1. *The figure shows, in panels A and B, the chemical shift perturbation (CSP) upon the addition of 0.1, 0.2, 0.4, 0.8, 1.2, 1.6, 2.6, and 5.2 equivalents of CLX4S to 1.0 equivalent of α-synuclein, as obtained from the 2D* ^{1}H-^{15}N *BEST-TROSY and 2D* ^{13}C-^{15}N *CON experiments, respectively. For the data reported in panel A and B, the protein concentration was 200 µM and 310 µM, respectively (in the buffer used in this work: 25 mM TRIS, 25 mM KCl, pH 7.2).*

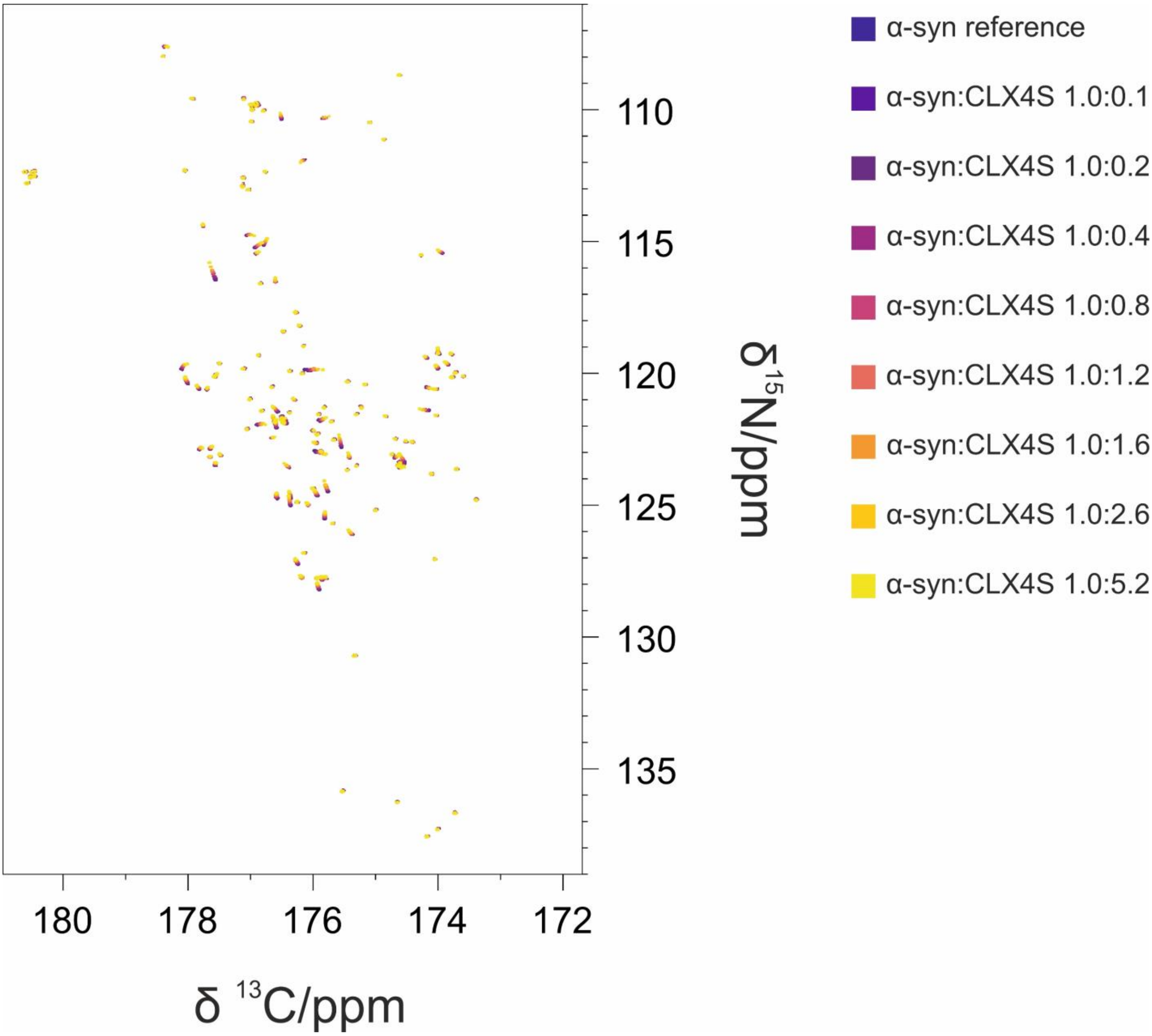

Figure S2: *The figure shows an overlay of the 2D* ^{13}C*-*^{15}N *CON NMR spectra acquired on α-synuclein in a concentration of 310 µM (blue) upon addition of CLX4S in the following α-synuclein: CLX4S molar ratios of 1.0:0.1 (dark violet), 1.0:0.2 (magenta), 1.0:0.4 (dark pink), 1.0:0.8 (pink), 1.0:1.2 (coral), 1.0:1.6 (orange), 1.0:2.6 (light orange), 1.0:5.2 (yellow).*

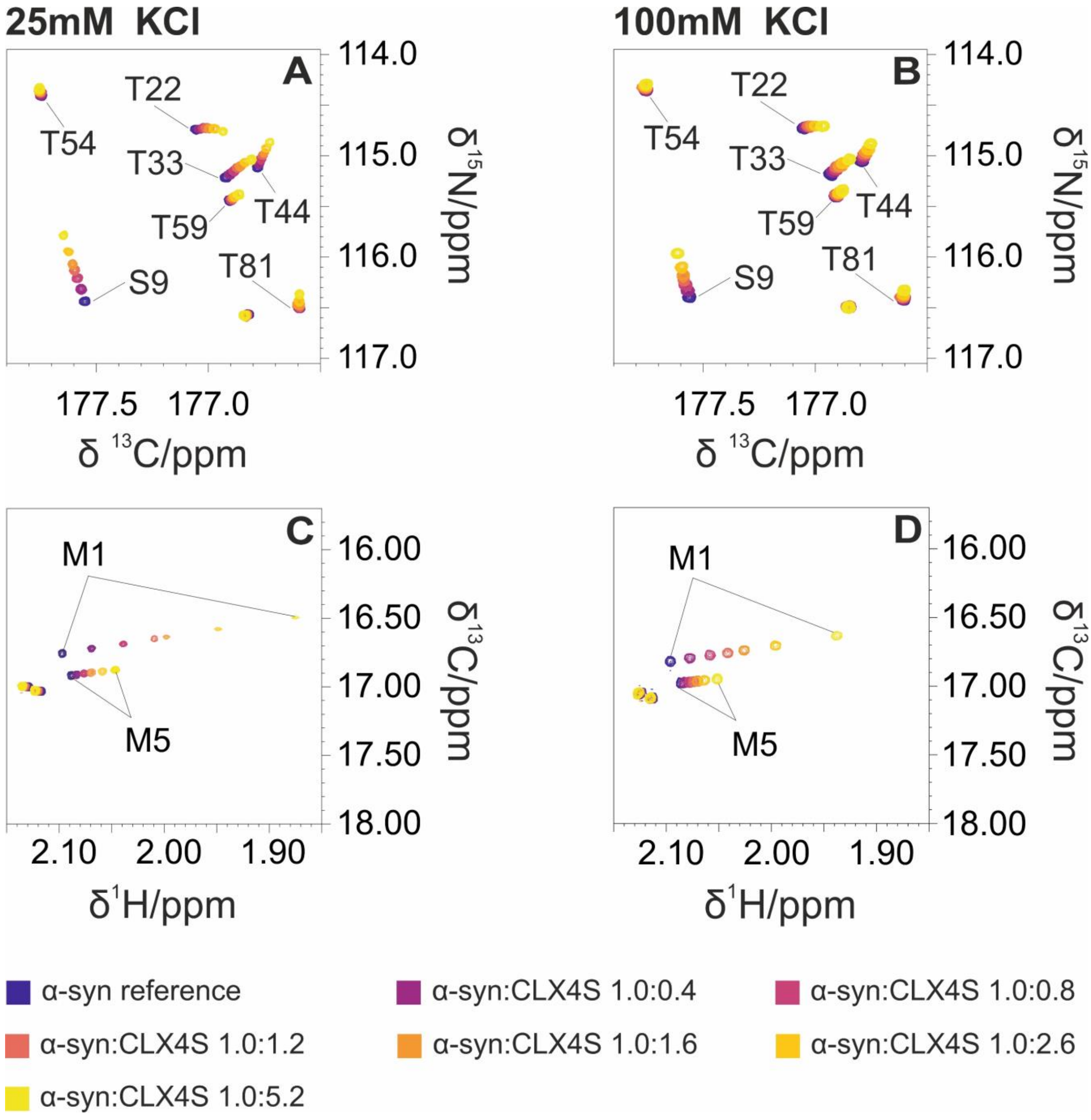

Figure S3. *The figure shows selected regions of the 2D ^{13}C-^{15}N CON spectra (panels A and B) and of the 2D ^{1}H-^{13}C methyl-HSQC (panels C and D) acquired upon addition of CLX4S to a solution containing 310 µM α-synuclein at different ionic strengths obtained by increasing the concentration of KCl in the buffer from 25 mM (panels A and C) to 100 mM (panels B and D). Each panel shows the overlay of the reference spectrum of α-synuclein (blue) with spectra acquired at α-synuclein:CLX4S molar ratios of 1.0:0.4 (dark pink), 1.0:0.8 (pink), 1.0:1.2 (coral), 1.0:1.6 (orange), 1.0:2.6 (light orange), 1.0:5.2 (yellow). The spectra were acquired at 298K on an instrument operating at 950 MHz (^{1}H Larmor frequency).*

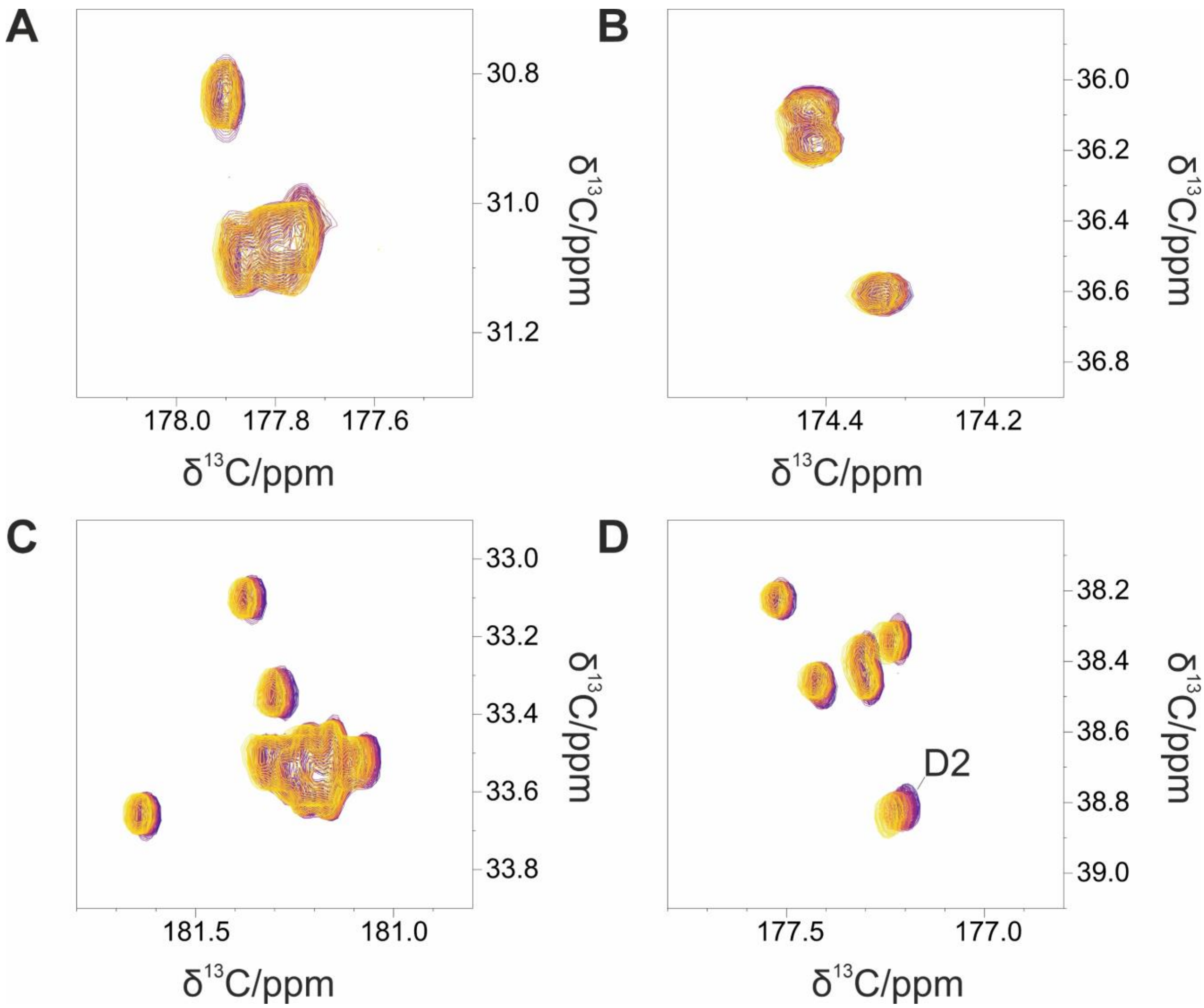

Figure S4. *The figure shows different regions of 2D CACO spectra reporting the resonances arising from side chain nuclear spins of glutamine (panel A), asparagine (panel B), glutamate (panel C) and aspartate (panel D) residues. The spectra show the overlay of the reference spectrum (310 µM α-synuclein sample) in blue with spectra acquired at α-synuclein:CLX4S molar ratios of 1.0:0.1, 1.0:0.2, 1.0:0.4, 1.0:0.8, 1.0:1.2, 1.0:1.6, 1.0:2.6, 1.0:5.2 (dark violet, magenta, dark pink, pink, coral, orange, light orange, yellow respectively).*

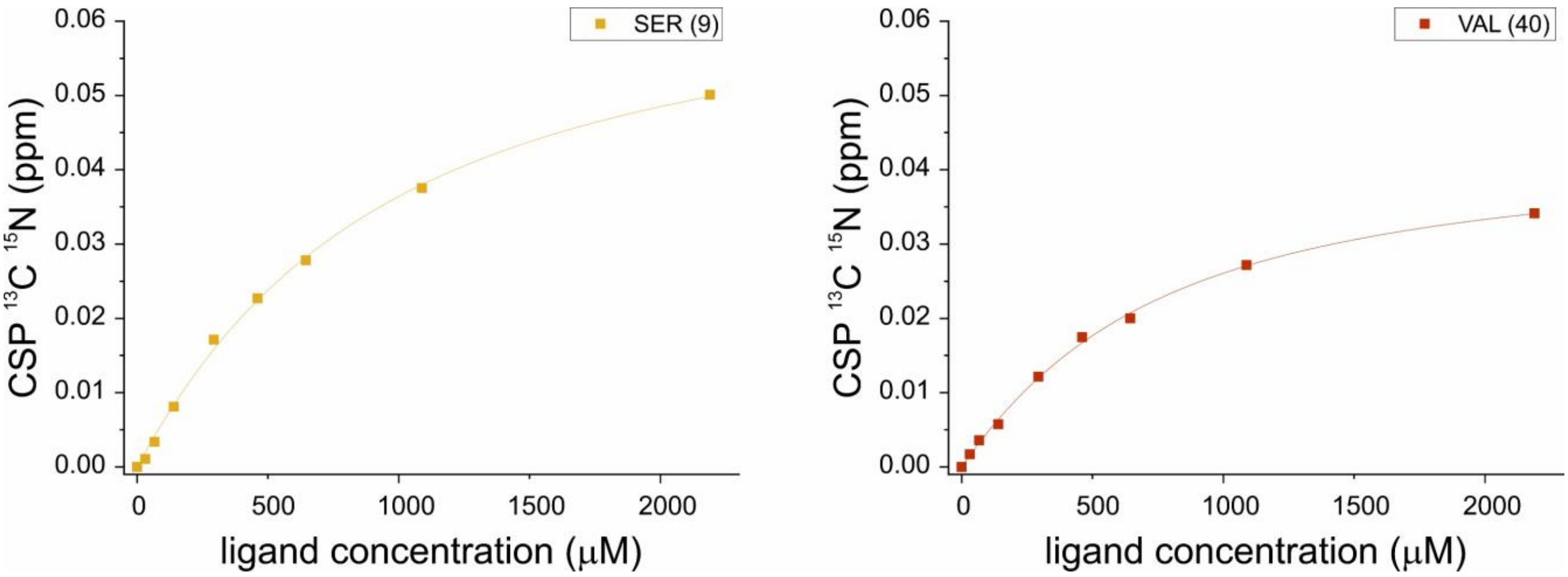

Figure S5. *The figure shows the fitting as obtained for S9 and V40 in yellow and red respectively. The obtained K_d for S9 and V40 are 740 ± 60 μM and 550 ± 40 μM respectively.*

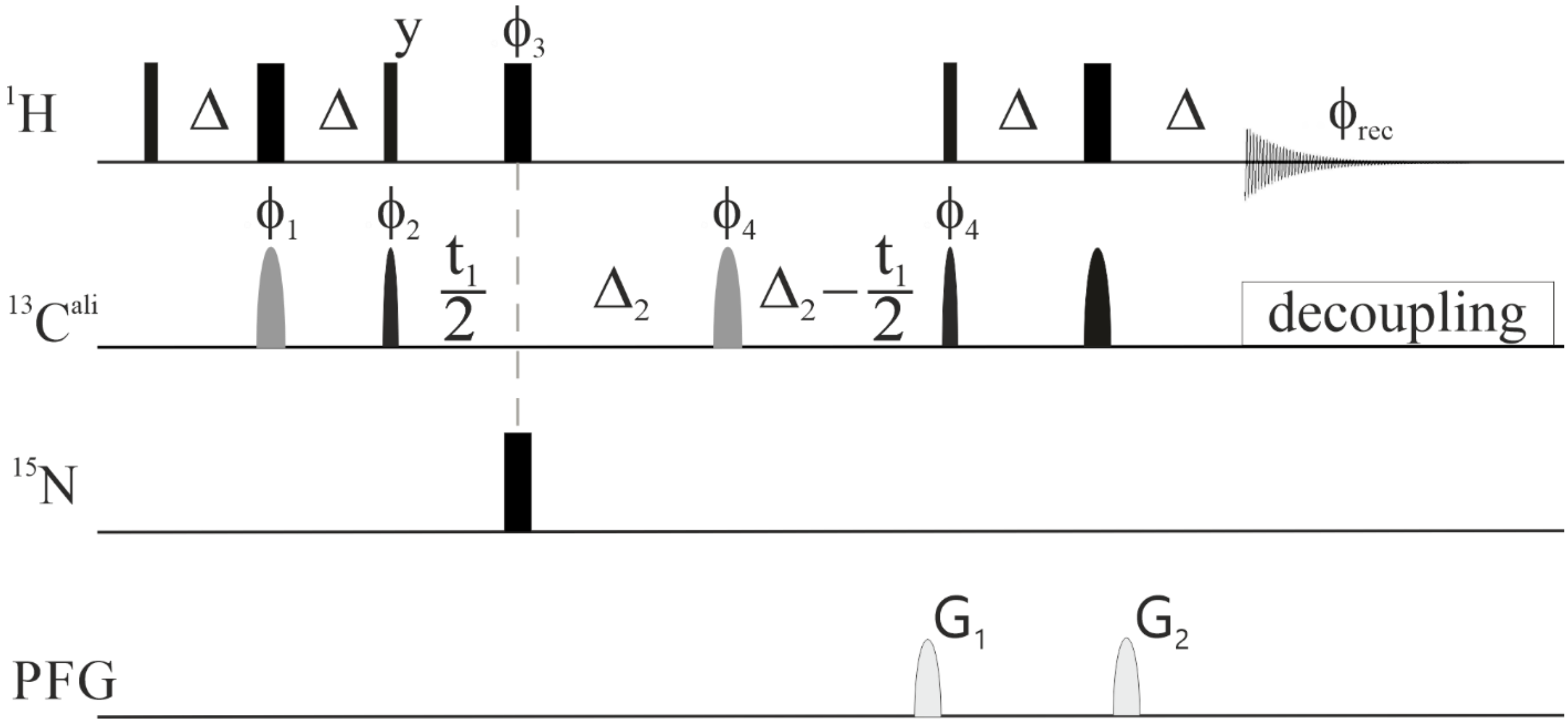

Figure S6. *Scheme of 2D ^{1}H-^{13}C methyl-HSQC pulse sequence tailored for the investigation of methyl resonances in IDPs. Narrow and wide black rectangles represent π/2 and π non-selective pulses; narrow and wide black bell shapes represent π/2 and π ^{13}C band-selective pulses for the methyl region. The wide grey bell shape represents a π pulse to cover the whole ^{13}C aliphatic region. The following phase cycling was employed: ϕ_1 = x; ϕ_2 = x, -x; ϕ_3 = 2(x), 2(-x); ϕ_4 = 4(x), 4(-x); ϕ_{rec} = x, -x, x, -x, -x, x, -x, x. Quadrature detection in the indirect dimensions was achieved through the echo-antiecho approach exploiting ϕ_1, ϕ_2 and ϕ_{rec} and gradient G_1. The length of the delays was: Δ = 1.92 ms; Δ_2 = 14.3 ms. Gradient durations and amplitudes were: G_1 = 1.00 ms 80%, G_2 = 1.00 ms 20.1%.*

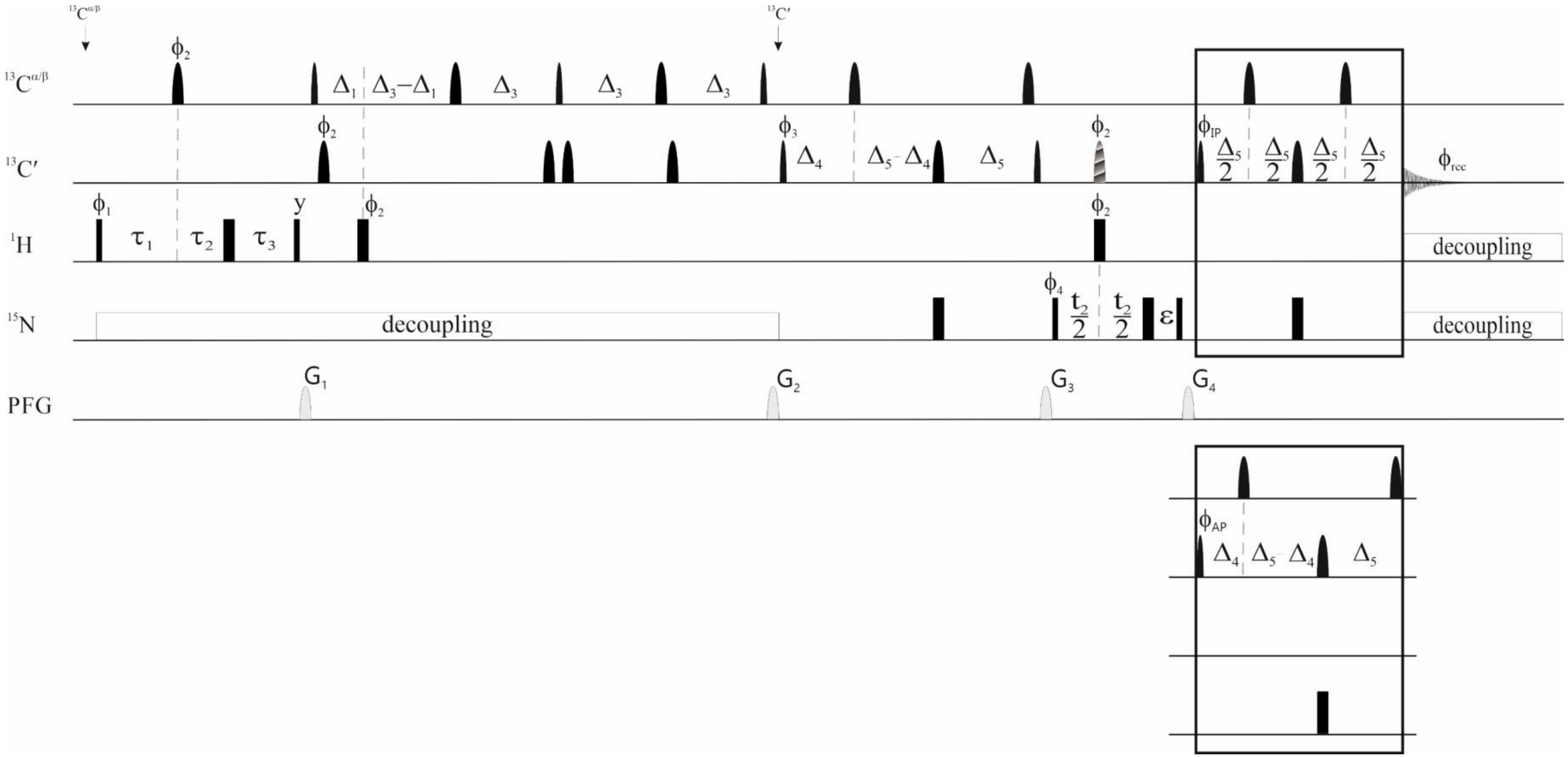

Figure S7. *Scheme of 3D HBHA(CBCA)CON pulse sequence. Narrow and wide black rectangles represent π/2 and π non-selective pulses; narrow and wide black bell shapes represent π/2 and π band-selective pulses. The wide striped bell shape represents the π pulse to cover both $^{13}C'$ and $^{13}C^{\alpha}$ resonances. The following phase cycling was employed: ϕ_1 = x, -x; ϕ_2 = 8(x), 8(-x) ϕ_3 = 4(x), 4(-x), ϕ_4 = 2(x), 2(-x); ϕ_{IP} = x; ϕ_{AP} = y; ϕ_{rec} = x, -x, -x, x, -x, x, x, -x. Quadrature detection in both the indirect dimensions was achieved through the States-TPPI approach incrementing ϕ_1 and ϕ_4. The length of the delays was: Δ = 1.8 ms; Δ_1 = 1.1 ms; Δ_3 = 4.0 ms; Δ_4 = 4.5 ms; Δ_5 = 15 ms; $\varepsilon = t_2(0) + p8$, where p8 is the duration of the adiabatic pulse on ^{13}C in the middle of the ^{15}N evolution period. ^{1}H (t_1) chemical shift evolution is performed using a semi-constant time period with delays $\tau_1 = \Delta + t_1/2$, $\tau_2 = (1-\Delta/2t_1^{max})t_1/2$, $\tau_3 = (1-t_1/t_1^{max})\Delta$. Gradients length and amplitudes were: G_1 = 1.00 ms 50%, G_2 = 1.00 ms 30%, G_3 = 1.00 ms 19%, G_4 = 1.00 ms 11%. The pulse sequence elements reported in the boxes represent the two variants to acquire either the in-phase (IP) or the antiphase (AP) components of carbonyl signals needed to achieve $^{13}C'$-$^{13}C^{\alpha}$ homonuclear decoupling through the IPAP approach[1].*

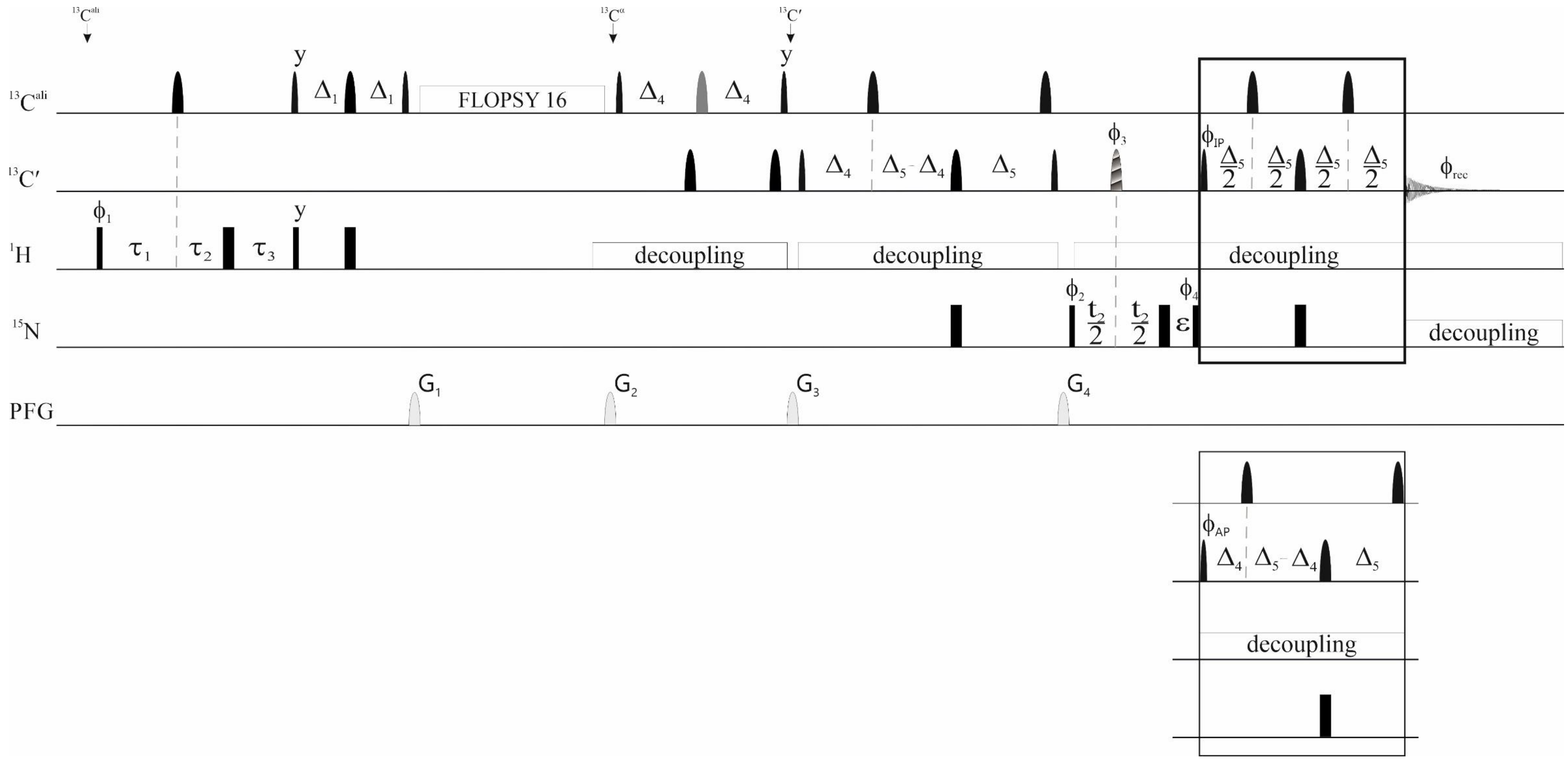

Figure S8. *Scheme of 3D H(CC)CON pulse sequence. Narrow and wide black rectangles represent π/2 and π non-selective pulses; narrow and wide black bell shapes represent π/2 and π band-selective pulses. The wide striped bell shape represents the π pulse to cover both $^{13}C'$ and $^{13}C^{\alpha}$ resonances. The following phase cycling was employed: ϕ_1 = x, -x; ϕ_2 = 2(x), 2(-x); ϕ_3 = 4(x), 4(-x); ϕ_4 = 8(x), 8(-x); ϕ_{IP} = x; ϕ_{AP} = -y ; ϕ_{rec} = x, -x, -x, x, x, -x, -x, x , -x, x, x, -x, -x, x, x, -x. Quadrature detection in both the indirect dimensions was achieved through the States-TPPI approach incrementing ϕ_1 and ϕ_2. The length of the delays was: Δ = 1.8 ms; Δ_1 = 0.95 ms; Δ_4 = 4.5 ms; Δ_5 = 12.5 ms; $\varepsilon = t_2(0) + p8$, where p8 is the duration of the adiabatic pulse on ^{13}C in the middle of the ^{15}N evolution period. ^{1}H (t_1) chemical shift evolution is performed using a semi-constant time period with delays $\tau_1 = \Delta + t_1/2$, $\tau_2 = (1-\Delta/2t_1^{max})t_1/2$, $\tau_3 = (1-t_1/t_1^{max})\Delta$. Gradients length and amplitudes were: G_1 = 0.60 ms 50%, G_2 = 0.60 ms 50%, G_3 = 1.00 ms 19%, G_4 = 1.00 ms 11%. The pulse sequence elements reported in the boxes represent the two variants to acquire either the in-phase (IP) or the antiphase (AP) components of carbonyl signals needed to achieve $^{13}C'$-$^{13}C^{\alpha}$ homonuclear decoupling through the IPAP approach*[1]

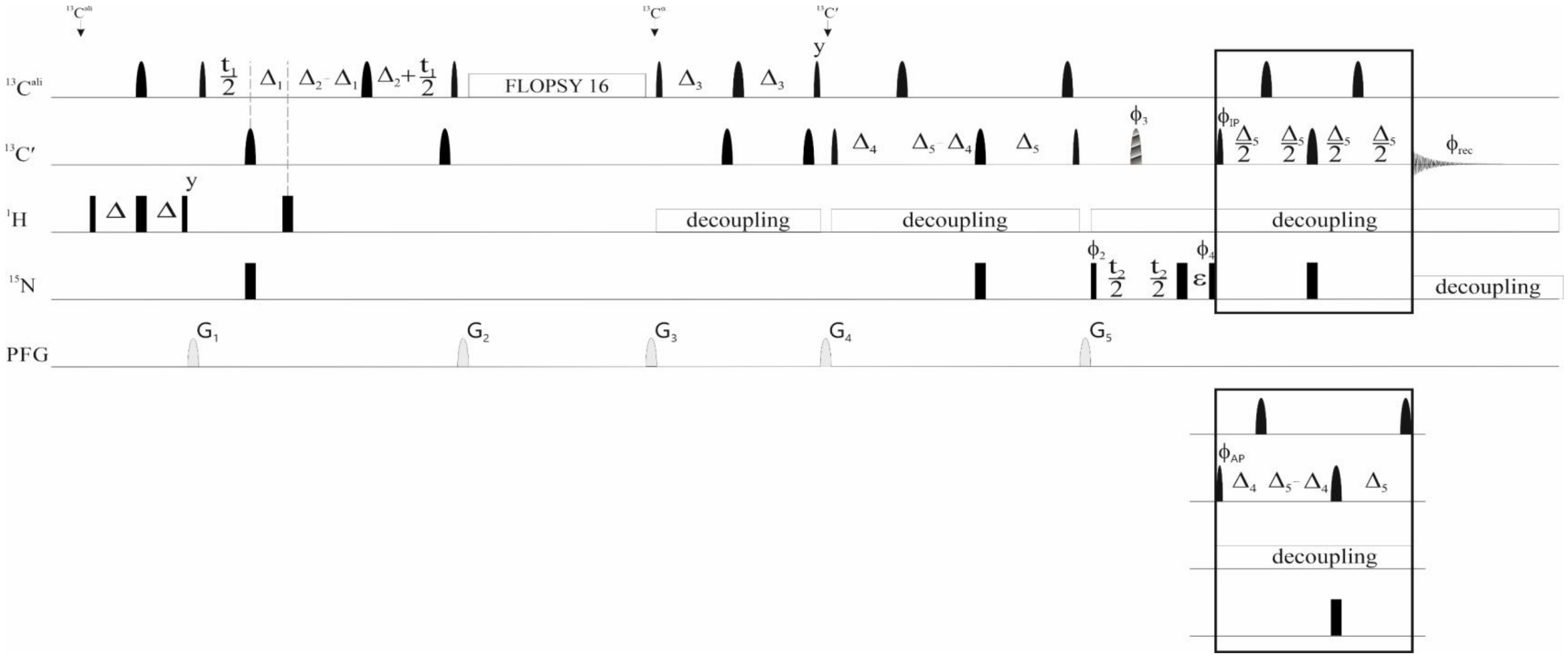

Figure S9. *Scheme of 3D CT (H)CCCON pulse sequence. Narrow and wide black rectangles represent π/2 and π non-selective pulses; narrow and wide black bell shapes represent π/2 and π band-selective pulses. The wide striped bell shape represents the π pulse to cover both $^{13}C'$ and $^{13}C^{\alpha}$ resonances. The following phase cycling was employed: ϕ_1 = y, -y; ϕ_2 = 2(x), 2(-x); ϕ_3 = 4(x), 4(-x); ϕ_4 = 8(x), 8(-x); ϕ_{IP} = x; ϕ_{AP} = -y; ϕ_{rec} = x, -x, -x, x, x, -x, -x, x, -x, x, x, -x, -x, x, x, -x. Quadrature detection in both the indirect dimensions was achieved through the States-TPPI approach incrementing ϕ_1 and ϕ_2. The length of the delays was: Δ = 1.8 ms; $Δ_1$ = 0.95 ms; $Δ_3$ = 4.0 ms; $Δ_4$ = 4.5 ms; $Δ_5$ = 12.5 ms; ε = $t_2(0)$ + p8, where p8 is the duration of the adiabatic pulse on ^{13}C in the middle of the ^{15}N evolution period. Gradients length and amplitudes were: G_1 = 1.00 ms 25%, G_2 = 0.60 ms 60%, G_3 = 0.60 ms 50%, G_4 = 1.00 ms 19%, G_5 = 1.00 ms 11%. The pulse sequence elements reported in the boxes represent the two variants to acquire either the in-phase (IP) or the antiphase (AP) components of carbonyl signals needed to achieve $^{13}C'$-$^{13}C^{\alpha}$ homonuclear decoupling through the IPAP approach*[1].

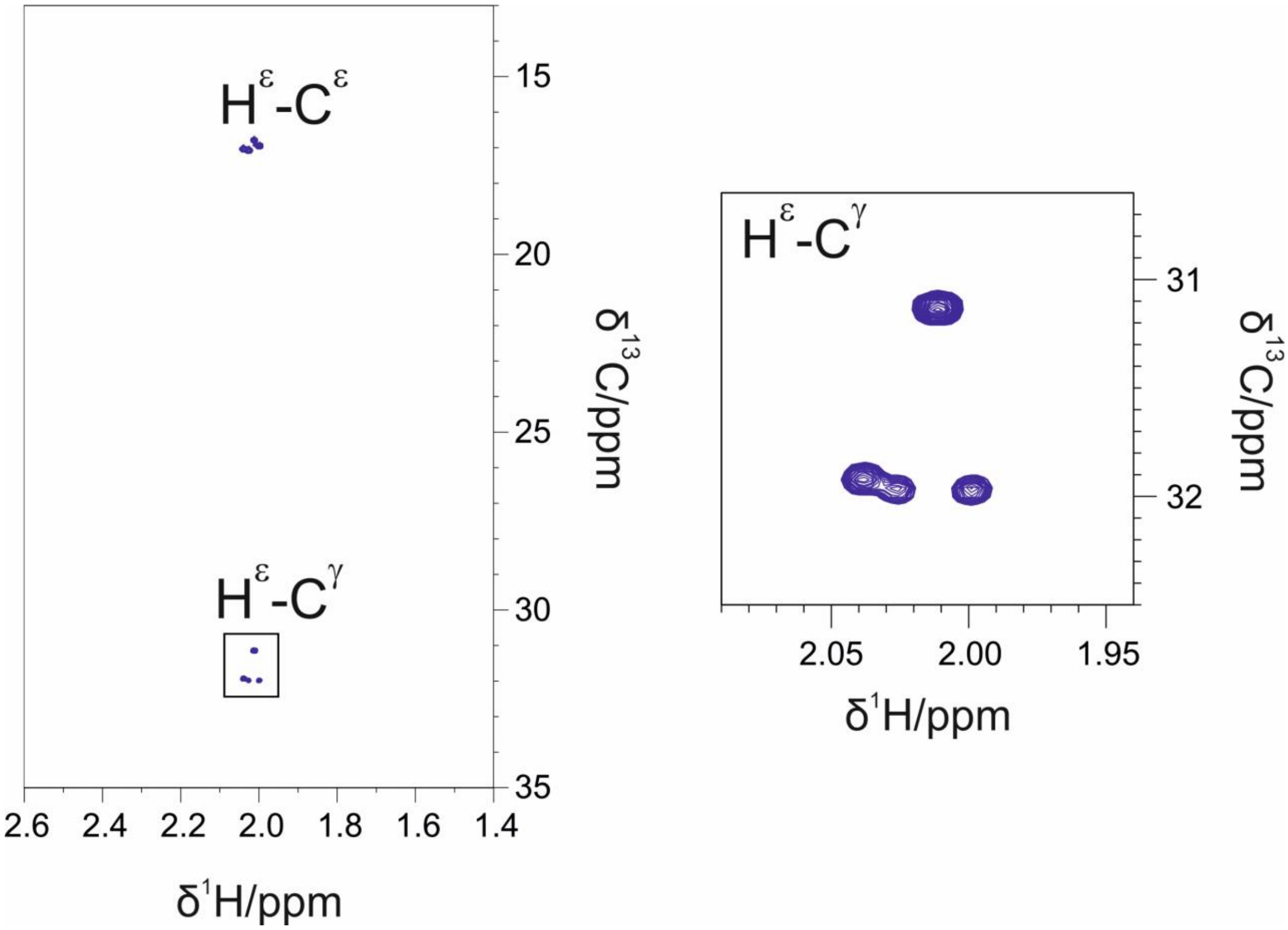

Figure S10: *The figure shows on the left the ^{1}H-^{13}C long range HSQC spectrum in the methionine region as obtained on α-synuclein. The correlations arising from both ^{1}J H$^{\varepsilon}$C$^{\varepsilon}$ and ^{3}J H$^{\varepsilon}$C$^{\gamma}$ coupling are visible. On the right, a zoom on the correlations arising from the ^{3}J H$^{\varepsilon}$C$^{\gamma}$ coupling are reported. Thanks to the previously obtained C$^{\gamma}$ assignment, the H$^{\varepsilon}$ and the C$^{\varepsilon}$ resonances can be derived in a sequence specific way.*

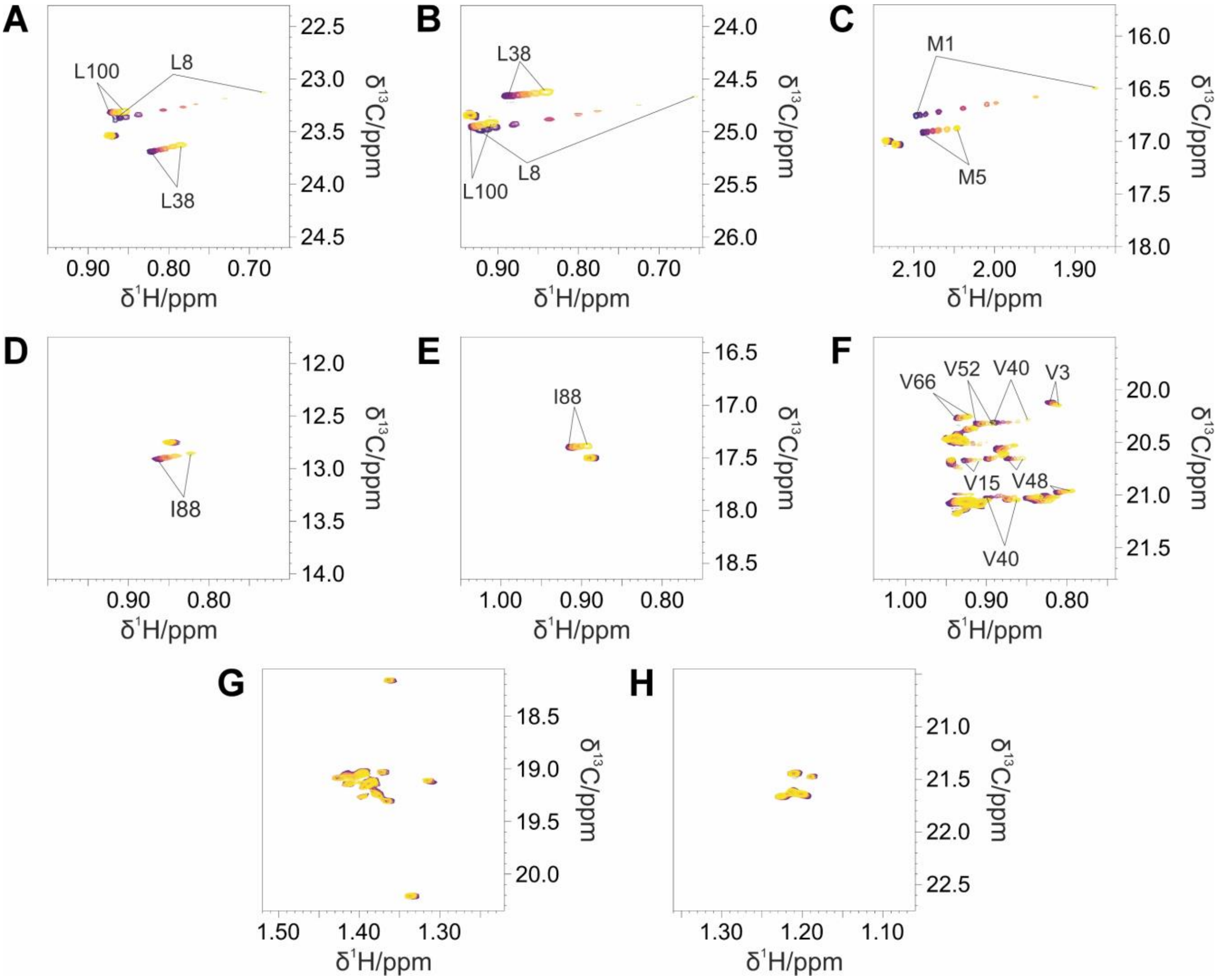

Figure S11. *The figure shows a close up on the different regions of the 2D ^{1}H-^{13}C methyl-HSQC spectrum acquired throughout the α-synuclein:CLX4S NMR titration. The panels show all the expected peaks arising from the methyl resonances (CH_3) of leucine (A, B), methionine (C), isoleucine (D and E), valine (F), alanine (G) and threonine (H) residues. Each panel shows the overlay of the reference spectrum (310 µM α-synuclein sample) with spectra acquired at α-synuclein: CLX4S molar ratios of 1.0:0.1 (dark violet), 1.0:0.2 (magenta), 1.0:0.4 (dark pink), 1.0:0.8 (pink), 1.0:1.2 (coral), 1.0:1.6 (orange), 1.0:2.6 (light orange), 1.0:5.2 (yellow). The spectra are shown using always the same spectral window and reporting the assignment for the most perturbed peaks.*

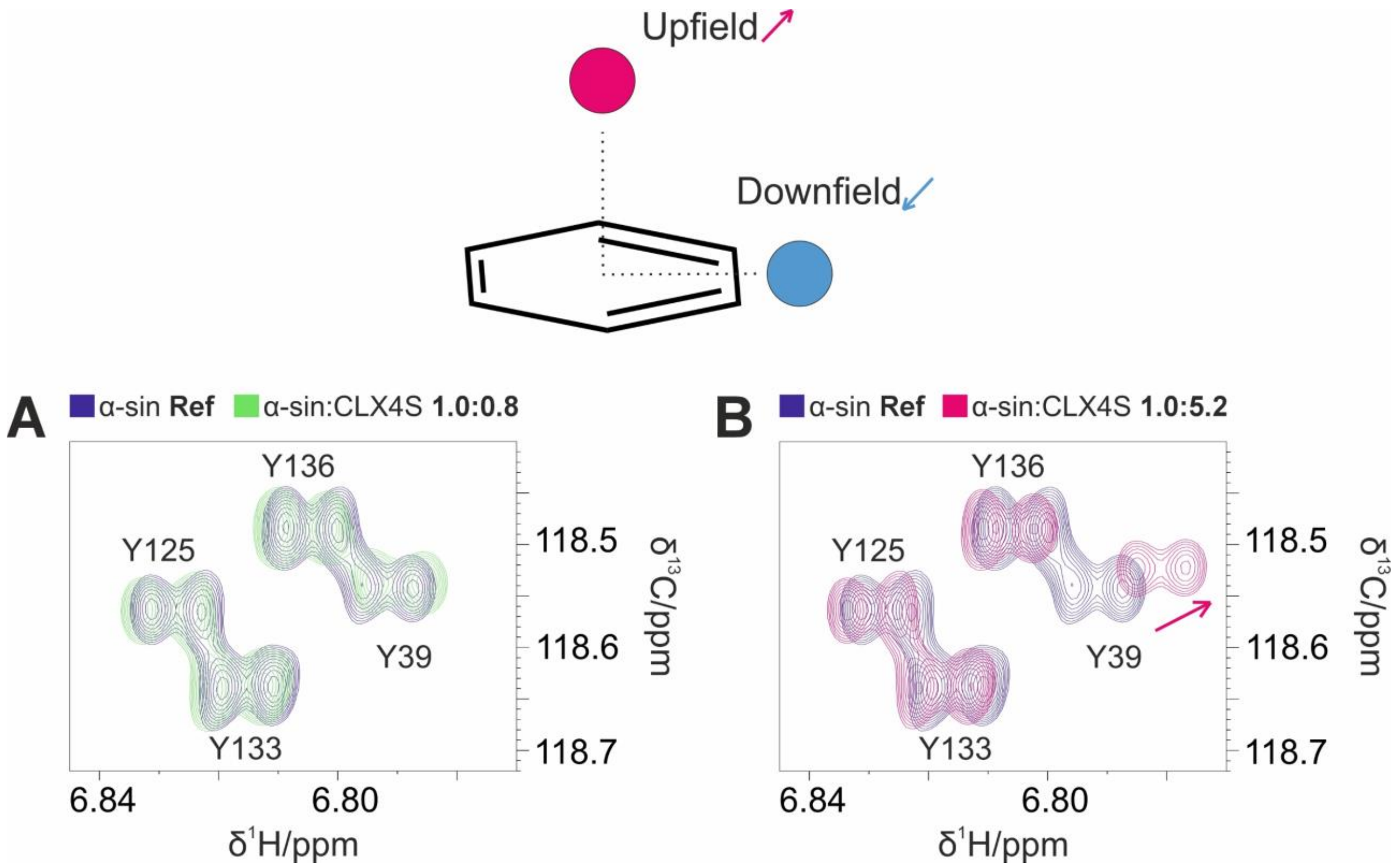

Figure S12. *^{1}H-^{13}C TROSY of aromatic side chains. Panels A and B show the superposition of the reference spectrum (310 µM α-synuclein sample in a buffer containing 25 mM TRIS, 100 mM KCl) in blue, and spectra acquired upon α-synuclein:CLX4S molar ratio of 1.0:0.8 and 1.0:5.2 in green and magenta respectively. The spectra were acquired at 298K on an instrument operating at 950 MHz (^{1}H Larmor frequency). A scheme with the expected chemical shift changes of a ^{1}H nuclear spin (spheres in the scheme) is reported on top of the figure depending on its relative position respect to the aromatic ring (in the same plane or perpendicular to the center of the aromatic ring).*

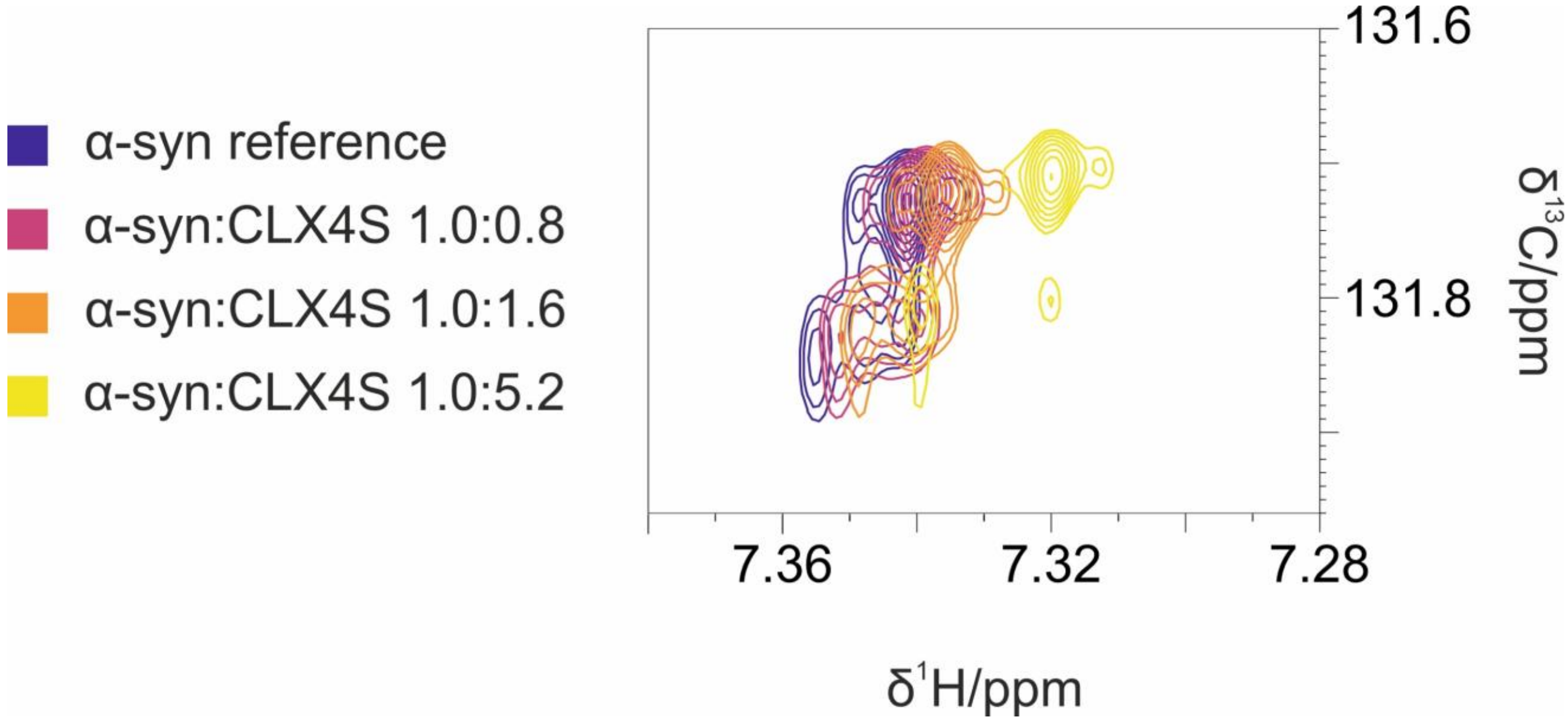

Figure S13. *^{1}H-^{13}C TROSY of aromatic sidechains in the region where the resonances of H^{ε}-C^{ε} of phenylalanine residues are found. The superposition of the reference spectrum (310 µM α-synuclein sample in a buffer containing 25 mM TRIS, 100 mM KCl) in blue and spectra acquired at α-synuclein:CLX4S in molar ratios of 1.0:0.8, 1.0:1.6, and 1.0:5.2 are reported in magenta, orange and yellow respectively.*

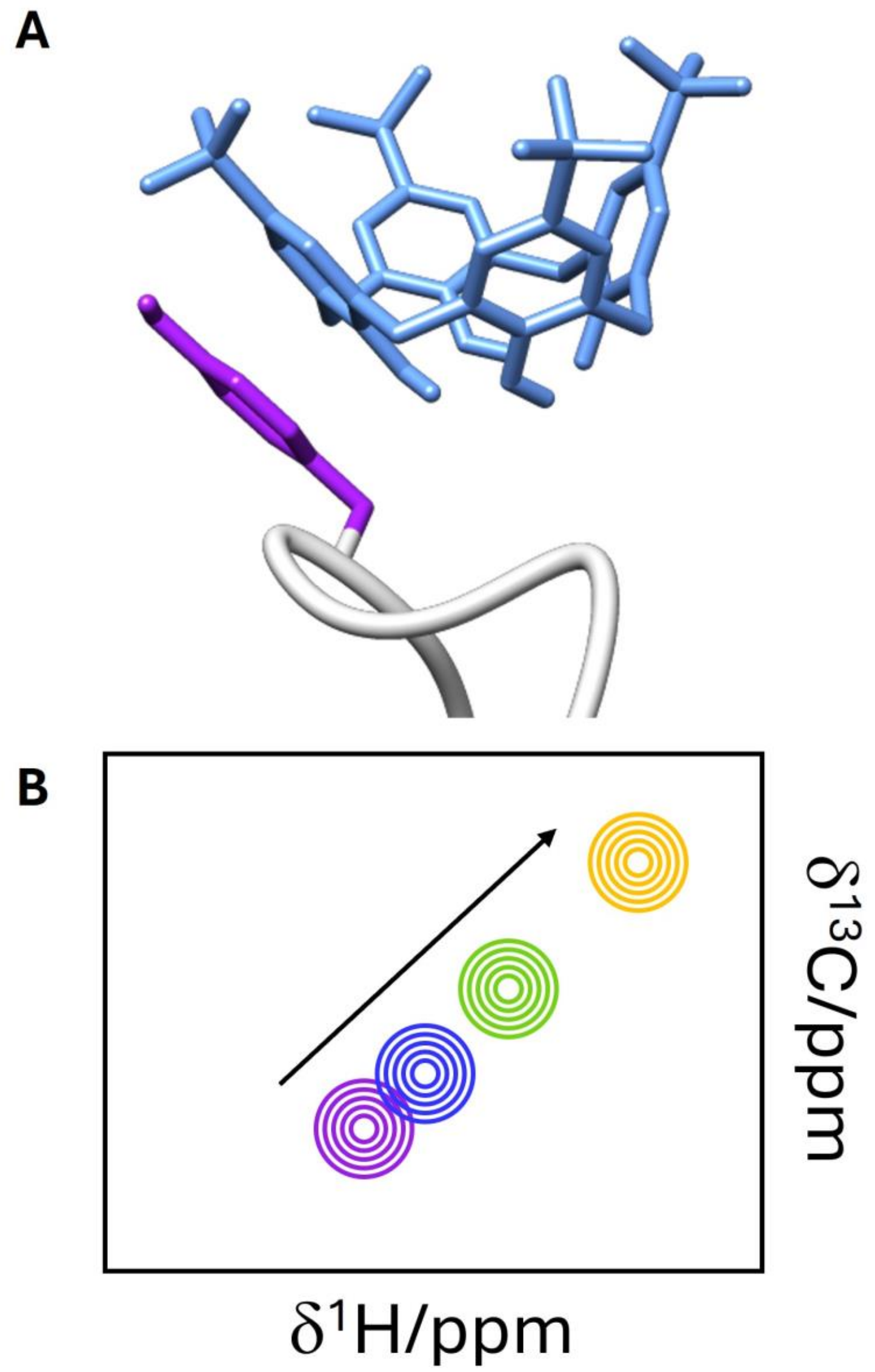

Figure S14. *Diagnostic upfiled shifts associated with CH-π and π-π interactions*[2,3] *illustrated using as an example a possible interaction mode between CLX4S and a tyrosine side chain from α-synuclein. In panel A, the planes of the two aromatic moieties (tyrosine side chain and one of the aromatic rings of CLX4S) are parallel one to another, possibly forming a π-π interaction, resulting in an upfield shift for the observed aromatic resonances (Panel B). CLX4S structure has been obtained from PDB entry 6EGY.*[4]

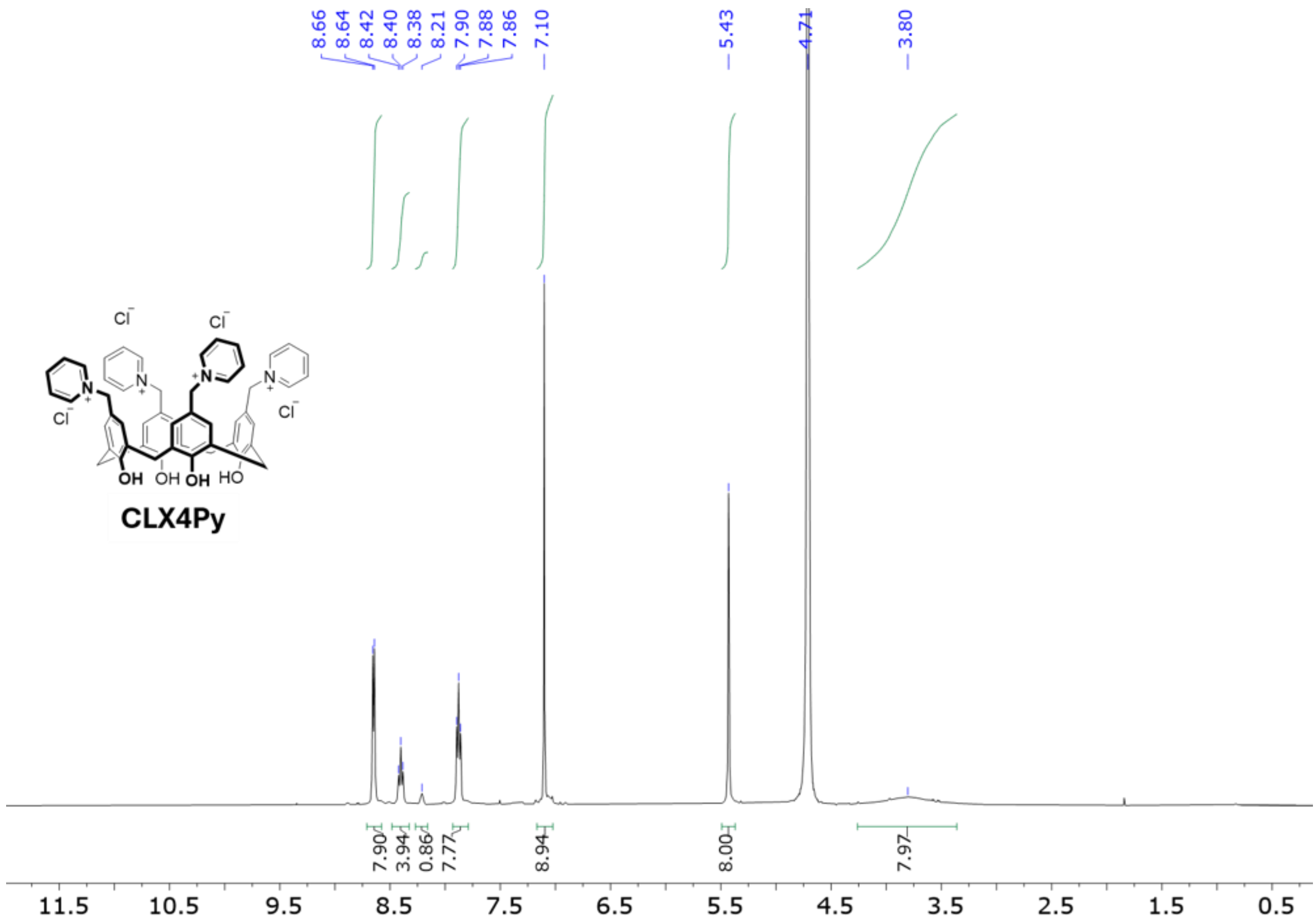

***Figure S15**: ^{1}H NMR spectrum of **CLX4Py** (400 MHz, D_2O).*

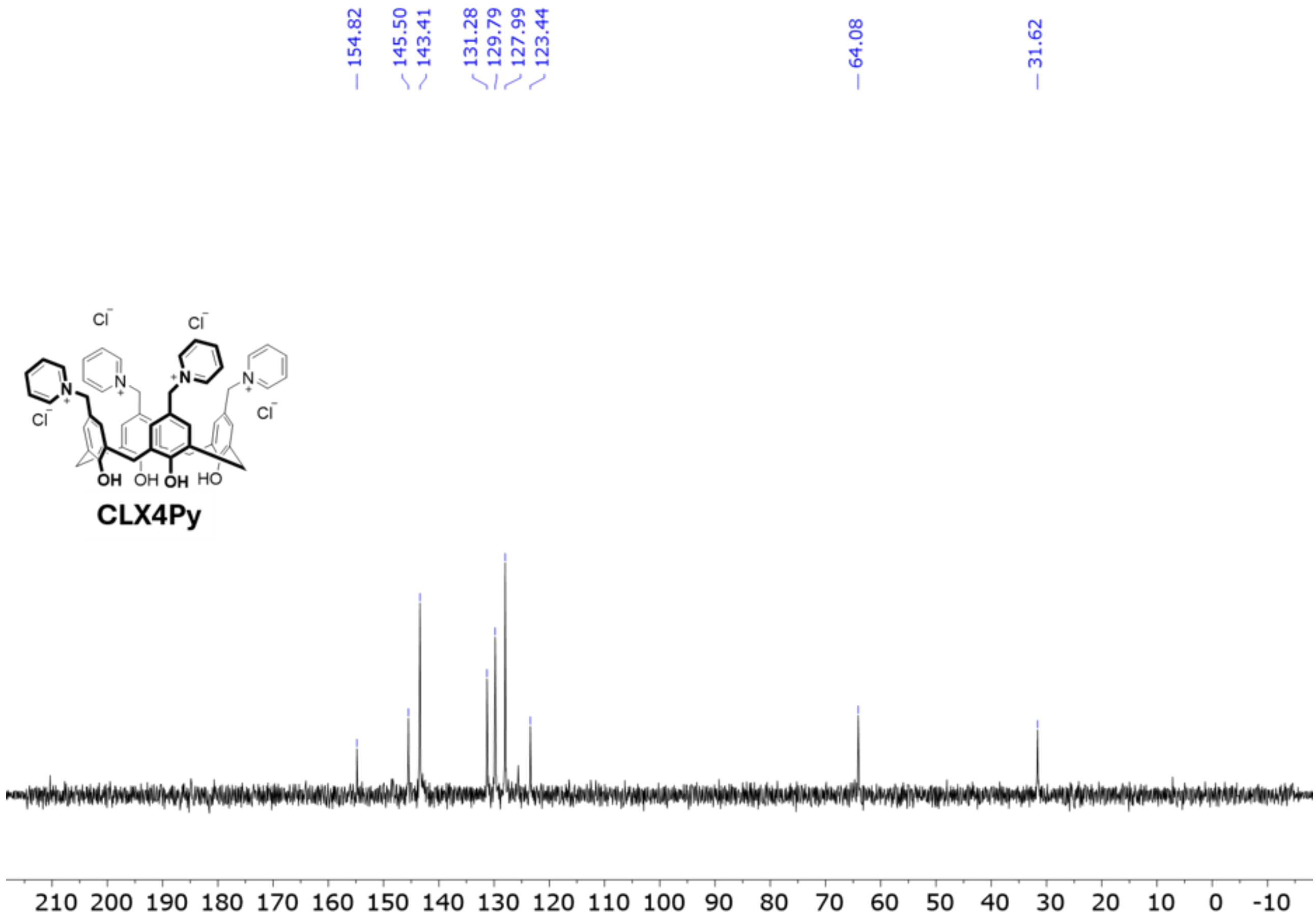

Figure S16: *^{13}C NMR spectrum of **CLX4Py** (400 MHz, D_2O).*

Supplementary References

(1) Felli, I. C.; Pierattelli, R. Spin-state-selective methods in solution- and solid-state biomolecular ^{13}C NMR. *Prog. Nucl. Magn. Reson. Spectrosc.* **2015**, *84–85*, 1–13. https://doi.org/10.1016/j.pnmrs.2014.10.001

(2) Beier, A.; Platzer, G.; Höfurthner, T.; Ptaszek, A. L.; Lichtenecker, R. J.; Geist, L.; Fuchs, J. E.; McConnell, D. B.; Mayer, M.; Konrat, R. Probing protein–ligand methyl–π interaction geometries through chemical shift measurements of selectively labeled methyl groups. *J. Med. Chem.* **2024**, *67* (15), 13187–13196. https://doi.org/10.1021/acs.jmedchem.4c01128

(3) Platzer, G.; Mayer, M.; Beier, A.; Brüschweiler, S.; Fuchs, J. E.; Engelhardt, H.; Geist, L.; Bader, G.; Schörghuber, J.; Lichtenecker, R.; Wolkerstorfer, B.; Kessler, D.; McConnell, D. B.; Konrat, R. PI by NMR: Probing CH–π interactions in protein–ligand complexes by NMR spectroscopy. *Angew. Chem. Int. Ed.* **2020**, *59* (35), 14861–14868. https://doi.org/10.1002/anie.202003732

(4) Mummidivarapu, V. V. S.; Rennie, M. L.; Doolan, A. M.; Crowley, P. B. Noncovalent PEGylation via sulfonatocalix[4]arene–a crystallographic proof. *Bioconjug. Chem.* **2018**, *29* (12), 3999–4003. https://doi.org/10.1021/acs.bioconjchem.8b00769
